# The Interaction of TRAF6 With Neuroplastin Promotes Spinogenesis During Early Neuronal Development

**DOI:** 10.3389/fcell.2020.579513

**Published:** 2020-12-09

**Authors:** Sampath Kumar Vemula, Ayse Malci, Lennart Junge, Anne-Christin Lehmann, Ramya Rama, Johannes Hradsky, Ricardo A. Matute, André Weber, Matthias Prigge, Michael Naumann, Michael R. Kreutz, Constanze I. Seidenbecher, Eckart D. Gundelfinger, Rodrigo Herrera-Molina

**Affiliations:** ^1^Laboratory of Synaptic Signaling, Department of Neurochemistry and Molecular Biology, Leibniz Institute for Neurobiology, Magdeburg, Germany; ^2^Division of Chemistry and Chemical Engineering, California Institute of Technology, Pasadena, CA, United States; ^3^Centro Integrativo de Biología y Química Aplicada, Universidad Bernardo O’Higgins, Santiago, Chile; ^4^Institute of Experimental Internal Medicine, Medical Faculty, Otto von Guericke University, Magdeburg, Germany; ^5^Leibniz Group ‘Dendritic Organelles and Synaptic Function’, Center for Molecular Neurobiology, University Medical Center Hamburg-Eppendorf, Hamburg, Germany; ^6^Center for Behavioral Brain Sciences, Magdeburg, Germany; ^7^Medical Faculty, Otto von Guericke University, Magdeburg, Germany

**Keywords:** dendritic protrusion, neuroplastin, E-I synapse balance, TRAF6, excitatory spinogenesis, synapse formation, neuronal connectivity

## Abstract

Correct brain wiring depends on reliable synapse formation. Nevertheless, signaling codes promoting synaptogenesis are not fully understood. Here, we report a spinogenic mechanism that operates during neuronal development and is based on the interaction of tumor necrosis factor receptor-associated factor 6 (TRAF6) with the synaptic cell adhesion molecule neuroplastin. The interaction between these proteins was predicted *in silico* and verified by co-immunoprecipitation in extracts from rat brain and co-transfected HEK cells. Binding assays show physical interaction between neuroplastin’s C-terminus and the TRAF-C domain of TRAF6 with a *K*_d_ value of 88 μM. As the two proteins co-localize in primordial dendritic protrusions, we used young cultures of rat and mouse as well as neuroplastin-deficient mouse neurons and showed with mutagenesis, knock-down, and pharmacological blockade that TRAF6 is required by neuroplastin to promote early spinogenesis during *in vitro* days 6-9, but not later. Time-framed TRAF6 blockade during days 6–9 reduced mEPSC amplitude, number of postsynaptic sites, synapse density and neuronal activity as neurons mature. Our data unravel a new molecular liaison that may emerge during a specific window of the neuronal development to determine excitatory synapse density in the rodent brain.

## Introduction

Synaptogenesis is a timely coordinated cellular process, which sets up the neuronal connectivity essential for information flow and processing in healthy brains (McAllister, 2007; Sudhof, 2008, 2017). Indeed, inaccuracy in synaptogenesis occurring massively during neuronal development in childhood is proposed as a critical factor in neuropsychiatric disorders including intellectual disability, autism spectrum disorders, and schizophrenia (Sudhof, 2008, 2017; Zhang et al., 2009; Boda et al., 2010; Caldeira et al., 2019). One key step in synaptogenesis is the massive appearance of spinogenic structures, named dendritic protrusions, in young dendrites, which differentiate into mature excitatory spine synapses. Protrusion formation seems to be controlled by molecules able to trigger spinogenic signaling mechanisms during a critical period in the neuronal development (Okawa et al., 2014; Jiang et al., 2017; Sudhof, 2017). Currently, there is limited knowledge on how such molecules organize the formation of primordial glutamatergic synapses and thus, it has not been fully appreciated how signaling events occurring during the development of neurons contribute to the establishment of future connectivity yielding correct synapse density in the brain (Yoshihara et al., 2009; Sudhof, 2017).

Neuroplastin is a type-1 transmembrane glycoprotein of the immunoglobulin superfamily of cell adhesion molecules (CAMs) (Langnaese et al., 1997; Beesley et al., 2014) shown to mediate the formation of a fraction of excitatory synapses in the hippocampus *in vivo* (Herrera-Molina et al., 2014; Amuti et al., 2016; Bhattacharya et al., 2017) and to belong to a group of highly expressed CAMs which define a “connectivity code” in the hippocampus during early postnatal development (Földy et al., 2016). Furthermore, neuroplastin has been identified as candidate to mediate the formation of synapses in the inner ear *in vivo* (Carrott et al., 2016). In mice, we have shown that constitutive elimination of neuroplastin expression goes along with autistic- and schizophrenic-like behaviors, altered brain activities, reduced synaptic plasticity, and unbalanced synaptic transmission (Bhattacharya et al., 2017; Herrera-Molina et al., 2017). Constitutive deficiency of neuroplastin expression results in lower numbers of excitatory synapses or abnormal synapse morphology in the mouse hippocampus (Herrera-Molina et al., 2014; Amuti et al., 2016). In contrast, inducible elimination of neuroplastin expression in fully developed adult mice does not modify the number of hippocampal excitatory synapses (Bhattacharya et al., 2017). However, it remains unknown how and when neuroplastin participates in synaptogenesis necessary for the proper establishment of synapse density, synaptic transmission, and neuronal activity.

The tumor necrosis factor (TNF) receptor-associated factor 6 (TRAF6) is essential for brain development as reduced programmed cell death in the diencephalon and mesencephalon resulted in lethal exencephaly in KO embryos (Lomaga et al., 1999). Moreover, TRAF6 has been closely related to pathologies of the central nervous system including traumatic brain injury, stroke and neurodegenerative diseases (for review see Dou et al., 2018). Furthermore, TRAF6 knockdown destabilizes PSD-95 and facilitates the plasticity of excitatory spines in mature neurons (Ma et al., 2017). Nevertheless, the functions of TRAF6 in young postnatal neurons, i.e., during the major period of excitatory synapse formation, are unknown. TRAF6 is a prominent adaptor protein with E3 ligase activity. It harbors an N-terminal RING domain followed by four zinc fingers and a C-terminal region that comprises a coiled coil domain and a TRAF-C domain (Chung et al., 2002; Yin et al., 2009). To initiate cell signaling in processes like neuroinflammation (Dou et al., 2018) as well as cell differentiation, activation and tolerance of immune cells, and migration of cancer cells (Lomaga et al., 1999; Kobayashi et al., 2001; Xie, 2013; Walsh et al., 2015), the TRAF-C domain docks the factor to a specific motif in cytoplasmic domains of transmembrane proteins allowing lateral homo-oligomerization of TRAF6 RING domains and assembly of a three-dimensional lattice-like structure (Yin et al., 2009; Ferrao et al., 2012; Wu, 2013). These TRAF6 structures are reported as plasma membrane-associated “fluorescent spots” on the micrometer scale where hundreds of cell signaling intermediaries would nest (Ferrao et al., 2012; Wu, 2013).

As we identified a TRAF6 binding motif in neuroplastin, but not in other known synaptogenic CAMs, we tested the hypothesis that TRAF6 interaction is required by neuroplastin for its capability to promote formation of excitatory synapses. This study uncovered a hitherto unanticipated function for TRAF6 in synaptogenesis during early neuronal development ultimately required for the adequate functioning of mature neurons.

## Materials and Methods

### Cells

Primary *Nptn*^–/–^ neurons were derived from hippocampi of *Nptn*^–/–^ mice (kindly provided by Dr. Dirk Montag, Leibniz Institute for Neurobiology), and compared to primary *Nptn*^+/+^ neurons derived from their proper control *Nptn*^+/+^ mice (Herrera-Molina et al., 2014; Bhattacharya et al., 2017). Co-cultures of rat hippocampal neurons and astrocytes were obtained as described (Herrera-Molina and von Bernhardi, 2005; Herrera-Molina et al., 2012). Human embryonic kidney (HEK) 293T cells were cultured as previously described (Herrera-Molina et al., 2017).

### DNA Constructs and Transfections

GFP-tagged neuroplastin constructs have been described (Herrera-Molina et al., 2017). Neuroplastin mutants flanked by HindIII and BamH1 restriction sites were generated from Np65-GFP plasmid by PCR amplification using the following primers for Np65-GFP forward: 5′-TCA AGC TTG CCA CCA TGT CG-3′ reverse: 5′-GGC GAT GGA TCC ATT TGT GTT TC-3′; Np65Δ-GFP reverse 5′-GGA TCC TGG CCT CTT CCT CTT CTC ATA C-3′: Np65_PED_-GFP forward 5′-GAG GAA GAG GGC AGA TGC GGT TCC TGC TG-3′ reverse 5′-CAG CAG GAA CCG CAT CTG CCC TCT TCC TC-3′. The mouse N-terminal Flag-tagged TRAF6 (Flag-TRAF6) mammalian expression plasmid was purchased from Addgene (#21624, GenBank: BAA12705.1). N-terminally GST-tagged TRAF6 (GST-TRAF6) and RING domain deficient TRAF6 with coiled-coil domain and TRAF6-C domain (289–530aa; GST-TRAF6_cc–c_) plasmids with BamH1 and EcoR1 restriction sites were generated by PCR amplification. GST-TRAF6 forward 5′-GAC AGG ATC CTC ATG AGT CTC TTA AAC-3′ reverse 5′-TAC GAA TTC CTA CAC CCC CGC ATC AGT A-3′; GST-TRAF6_cc–c_ forward 5′-GCG TCG GAT CCA TAT GGC CGC CTC T-3′; TRAF6-GFP forward 5′-GTG AAG CTTCTA ATG AGT CTC TTA AAC TGT GA-3′ reverse 5′-ATA AGG ATC CCT ACA CCC CCG CAT C-3′; TRAF6_cc–c_-GFP forward 5′-GTG AAG CTT CTA ATG GCC GCC TCT-3′. Scrambled siRNA (sc-37007) and TRAF6 siRNA (sc-36717) were purchased from Santa Cruz. HEK cells and primary neuronal cultures were transiently transfected with plasmid DNA constructs using Lipofectamine 2000 (Invitrogen/ThermoFisher) in optiMEM media (Gibco). Neurons were transfected with scrambled siRNA or TRAF6 siRNA (30 nM) using siLentFect (Bio-Rad) at 6 DIV.

### *In silico* Modeling

We performed local peptide docking based on interaction similarity and energy optimization as implemented in the GalaxyPepDock docking tool (Lee et al., 2015). The protein–peptide complex structure of the hTRANCE-R peptide bound to the TRAF6 protein as provided by Ye et al. (2002) was used as input (PDB: 1LB5). The docking employs constraints of local regions of the TRAF6 surface based on the interaction template. The energy-based optimization algorithm of the docking tool allows efficient sampling of the backbone and side-chains in the conformational space thus dealing with the structural differences between the template and target complexes. Models were sorted according to protein structure similarity, interaction similarity, and estimated accuracy. The fraction of correctly predicted binding motif residues and the template-target similarity was used in a linear model to estimate the prediction accuracy. The model using target-template interactions based on the QMPTEDEY motif of the hTRANCE-R template was selected (TM score: 0.991; Interaction similarity score 108.0; Estimated accuracy: 0.868).

### Surface Plasmon Resonance

Protein–Protein interaction measurements were carried out on a BIACORE X100 (GE Healthcare Life Sciences). Sensorgrams were obtained as single cycle kinetics runs. Therefore, increasing concentrations of neuroplastin peptide (2.5, 5, 100, 200, and 400 μM) or just running buffer (startup) were sequentially injected on GST-TRAF6 coated CM5 sensor chip (GE). Unspecific bindings were calculated by using a GST-coated sensor as reference response. Immobilization of these proteins was done using the amine coupling kit as we described in Reddy et al. (2014). All runs were performed in HBS-P buffer. Analysis of affinity was performed using the BIACORE X100 Evaluation Software 2.0.1 (Reddy et al., 2014).

### GST Pull-Down Assay

GST, GST-TRAF6 and GST-TRAF6_cc–c_ were transformed into *Escherichia coli* BL21 (DE3) bacterial strain and induced by 0.5 mM of isopropyl-1-thio-*b*-D-galactopyranoside (IPTG) for 6 h at 25°C. The cells were lysed in resuspension buffer [50 mM Tris-HCl pH 8.0, 150 mM NaCl and protease inhibitor cocktail (Roche)] with sonication on ice. The purifications of these proteins from transformed bacterial cell extract were performed according to manufacturer instructions (GST bulk kit, GE Healthcare Life Sciences). The purified soluble GST proteins were immobilized on glutathione sepharose 4B beads (GE Healthcare Life Sciences). The beads were washed with binding buffer at least four times, and the pull-down samples were subsequently subjected to immunoblot analyses. The 5 μg of fusion protein coupled beads (GST, GST-TRAF6 and GST-TRAF6_cc–c_) were incubated with lysate from HEK cells transfected with Np65-GFP for 1 h at 4°C in 500 μl RIPA lysis buffer. The beads were washed and eluted with pre-warmed SDS sample buffer. The eluted proteins were resolved by SDS-PAGE.

### Co-immunoprecipitation Assays

HEK cells overexpressing GFP-tagged constructs were washed in ice-cold PBS and lysed using radioimmunoprecipitation assay (RIPA) buffer contained 20 mM of Tris (pH 7.5), 100 mM of NaCl, 1 mM EDTA, 10% glycerin, 0.1% SDS, 1% Triton X-100, 1 mM AEBSF, 1 mM sodium orthovanadate, 1 mM sodium molybdate, 1 mM *N*-Ethylmaleimide, 20 mM sodium fluoride, 20 mM glycerol-2-phosphate, 10 mM potassium hydrogen phosphate, 10 mM sodium pyrophosphate and protease inhibitor cocktail (Roche). Samples were incubated with GFP antibody-coupled magnetic beads (μMACS) at 4°C for 4 h. Immunoprecipitated complexes were eluted using μMACS GFP isolation kit (#130-091-125) according to manufacturer’s instructions. Eluted proteins were subjected SDS-PAGE.

Hippocampus from 2 weeks-old *Nptn*^+/+^ and *Nptn*^–/–^ mice or forebrains of 3 weeks-old rats were stored at –80°C until use. After homogenization in ice-cold RIPA buffer, which preserves strong protein-protein interactions (Müller et al., 1996; Lin et al., 1998), supplemented with and protease inhibitor cocktail (Roche) at 4°C, total homogenates were precleared by 30 min incubation with Protein G Sepharose TM 4 Fast Flow (GE Healthcare) and then incubated overnight with a rabbit anti-neuroplastin antibody that recognized the Ig-like domain 2 and 3, which are common for Np65 and Np55 (1 μg/ml, Smalla et al., 2000; Bhattacharya et al., 2017). Precipitation was performed by adding Protein G Sepharose beads for 2 h at 4°C. Beads were washed ones in RIPA buffer, two times in 20 mM Tris, 150 mM NaCl, 0.5% Digitonin, pH 7.5 followed by a short rinse in 20 mM Tris/150 mM NaCl. For SDS-PAGE, bound proteins were eluted with 1x Rotiload (Roth). Eluted proteins were subjected to SDS-PAGE.

### Immunoblot Analysis

Proteins were separated by sodium dodecyl sulfate–polyacrylamide gel electrophoresis (SDS-PAGE) on 10% gels and transferred to a nitrocellulose membrane (Whatman). After blocking with 5% non-fat milk in Tris-buffered saline (TBS) containing 0.1% of Tween 20 for 1 h, the membranes were incubated with indicated antibodies overnight, washed with TBS three times, and then incubated with corresponding secondary antibody conjugated to horseradish peroxidase enzyme for 1 h. Immunodetection was performed with the following antibodies: anti-Flag mouse (Sigma, #F1804; 1:2,000), anti-GFP rabbit (Abcam, #ab290; 1:2,500), anti-TRAF6 mouse (Santa Cruz, #sc-8709; 1:1,000) and anti-β-actin mouse (Sigma, #A5441; 1:1,000), horseradish peroxidase-conjugated anti-mouse (Dako, #P0447; 1:4,000) or anti-rabbit IgG (gamma-chain specific, Sigma, #A1949-1VL; 1:4,000) antibodies.

### Immunocytochemistry

Hippocampal neurons were fixed with cold methanol and then washed with a solution containing 10% horse serum, 0.1 mM glycine, and 0.1% Triton X-100 in Hanks’ balanced salt solution four times for 5 min. Fixed samples were incubated with indicated primary antibodies for overnight at 4°C. To visualize dendritic protrusions, after transfection, pyramidal neurons were morphologically identified based on the side and shape of cell body as observed using anti-MAP2 guinea pig (Synaptic Systems, #188 004; 1:1,000) and anti-GFP mouse (Sigma Aldrich, #11814460001; 1:1,000) antibodies. Routinely, neuron identity was confirmed using an anti-Ctip2 rat (Abcam, #25B6; 1:250) (Herrera-Molina et al., 2014). Subsequently, samples were incubated with anti-guinea pig Cy5-, anti-mouse Alexa 488-conjugated secondary antibodies (1:1,000) generated in donkey (Jackson ImmunoResearch) for 1 h at RT. Other primary antibodies used were: anti-TRAF6 rabbit (Santa Cruz, #sc-7221; 1:100), anti-Synapsin 1 rabbit (Synaptic Systems, #106 103; 1:500), anti-Shank2 guinea pig antibody (Synaptic Systems, #162 204; 1:1,000), anti-Homer1 mouse (Synaptic Systems, #160 011; 1:500); anti-MAP2 guinea pig (Synaptic Systems, #188 004; 1:1,000) primary antibodies for overnight at 4°C. Subsequently, samples were incubated with anti-rabbit 405-, anti-mouse Cy5-, anti-rat Alexa 488- and/or anti-guinea pig Cy3-conjugated donkey secondary antibodies (1:1,000) for 1 h. Then samples were washed and mounted with Mowiol. Quantification of synapse marker signals was performed as in detail described in Herrera-Molina et al. (2014).

### Image Acquisition and Processing and Co-localization

Images were acquired using HCX APO 63/1.40 NA or 100/1.4NA objectives coupled to a TCS SP5 confocal microscope under sequential scanning mode with 4.0- to 6.0-fold digital magnification. Z-stacks with 41.01 × 41.01 × 5 μm physical lengths were digitalized in a 512 × 512 pixels format file or with 61.51 × 15.33 × 2 μm in a 1024 × 256 pixel format file. To correct optical aberrations, z-stack images were deconvolved using the Huygens Professional software v. 19.10 (Scientific Volume Imaging B.V., Netherlands). Pearson’s co-localization index from single z-planes was obtained from dendritic segments containing dendritic protrusions using Imaris software v. x64 9.5.1 (Bitplane Scientific Software, Oxford Instruments plc).

### Quantification of Filopodia and Dendritic Protrusions

In HEK cells, filopodia number and length were quantified using a MATLAB-based algorithm, FiloDetect, with some modifications (Nilufar et al., 2013). The algorithm was run for every single image, and the image threshold was adjusted to avoid false filopodia detection and to quantify precise filopodia length and number. The filopodia number per μm was calculated from perimeter of the cell using ImageJ. In neurons, the dendritic protrusions were quantified manually using maximum intensity and Z-projection method of ImageJ software. The dendritic protrusions were considered between 0.25 and 20 μm length. Shank2 clusters were quantified from manually cropped images using brightness-enhanced original GFP fluorescent as reference to identify puncta of interest. For this, Shank2 clusters overlapping with GFP fluorescence were obtained by using the “image calculator” command in ImageJ. Regions of interest (the dendritic protrusions) were defined according to GFP fluorescence with polygon selection tool. Images were processed with watershed segmentation to refine the shapes of Shank2-positive objects in binary images. The area, intensity and number of Shank2 clusters in dendritic protrusions were measured by filtering the cluster size (minimum 0.02 μm^2^) using ImageJ software as further detailed in Herrera-Molina et al. (2014).

### Synaptotagmin Uptake Assay

Presynaptic activity driven by endogenous network activity was monitored as described before (Herrera-Molina et al., 2014). Hippocampal neurons were washed once with pre-warmed Tyrodes solution (119 mM NaCl, 2.5 mM KCl, 25 mM HEPES, pH 7.4, 30 mM glucose, 2 mM MgCl_2_, 2 mM CaCl_2_) and immediately incubated with an Oyster 550-labeled anti-synaptotagmin-1 rabbit antibody (Synaptic Systems, #105 103C3; 1:500) for 20 min at 37°C. After the antibody uptake, neurons were washed, fixed, and stained with anti-VGAT guinea pig (Synaptic Systems, #131 004; 1:1,000) and anti-synaptophysin mouse (company, catalog number; 1:1,100) primary antibodies overnight at 4°C. Subsequently, samples were incubated with anti-rabbit Cy3-, anti-guinea pig Cy5- and anti-mouse Alexa 488-conjugated donkey secondary antibodies (1:1,000) for 1 h. Z-stack images of soma and secondary/tertiary dendrites were acquired using an oil-immersion (HCX APO 63/1.40 NA) objective coupled to a TCS SP5 confocal microscope under sequential scanning mode with a 4.0-fold digital magnification, and digitalized in a 512 × 512 pixels format file (61.51 × 61.51 μm physical lengths). All parameters were rigorously maintained during the image acquisition. For quantification, *z*-stacks were projected using “sum slices” Z-projection method of ImageJ software. We quantified the synaptotagmin-associated fluorescence co-localizing with 1-bit masks derived from VGAT-positive (inhibitory presynapses) or VGAT-negative synaptophysin-positive (excitatory presynapses) puncta using the “image calculator” in the ImageJ software. During image processing the original settings of the synaptotagmin channel were carefully maintained as the original. One-bit masks were generated using the analyze particle in the ImageJ software for a segmented image of each presynaptic marker (range of particle size 0.15 – 2.25 μm^2^ for inhibitory presynapses and 0.15 – 1.50 μm^2^ for excitatory presynapses).

### Electrophysiology

Whole-cell patch clamp recordings were performed under visual control using phase contrast and sCMOS camera (PCO panda 4.2). Borosilicate glass pipettes (Sutter Instrument BF100-58–10) with resistances ranging from 3 to 7 MΩ were pulled using a laser micropipette puller (Sutter Instrument Model P-2000). Electrophysiological recordings from neurons were obtained in Tyrodes solution ([mM] 150 NaCl, 4 KCl, 2 MgCl_2_, 2 MgCl_2_, 10 D-glucose, 10 HEPES; 320 mOsm; pH adjusted to 7.35 with NaOH and Osmolarity of 320 mOsm) + 0.5 μM TTX (Tocris). Pipettes were filled using standard intracellular solution ([mM] 135 K-gluconate, 4 KCl, 2 NaCl, 10 HEPES, 4 EGTA, 4 MgATP, 0.3 NaGTP; 280 mOsm; pH adjusted to 7.3 with KOH). Whole-cell configuration was confirmed via increase of cell capacitance. During voltage clamp experiments neurons were clamped at –70 mV. Whole-cell voltage clamp recordings were performed using a MultiClamp 700B amplifier, filtered at 8 kHz and digitized at 20 kHz using a Digidata 1550A digitizer (Molecular Devices). Data were acquired and stored using Clampfit 10.4 software (HEKA Electronics) and analyzed with Mini-Analysis (Synaptosoft Inc., Decatur, GA, United States). The neuronal activity from 200.000 hippocampal cells was sampled extracellularly at 10 kHz using MC_Rack software and MEA1060INV-BC system (MultiChannel Systems, Reutlingen, Germany) placed inside of a cell culture incubator in order to provide properly controlled temperature, humidity, and gas composition as described (Bikbaev et al., 2015). The recordings were initiated after a resting period of 30 min after physical translocation of each individual MEAs to the recording system. The off-line analysis was carried out on 600-s long sessions per MEA at each experimental condition. The detection of spikes was performed after a high-passed (300 Hz) filtering and processing of signals and analyses of neuronal activity were carried out using Spike2 software (Cambridge Electronic Design, Cambridge, United Kingdom).

### Statistical Analysis

For statistical analysis, Prism 5 software (GraphPad) was used. The results are presented as mean ± SEM (standard error of the mean). The *n* number of cells or N individual experiments or samples as well as statistical tests used to evaluate significant differences are given in the figure legends.

## Results

### A TRAF6 Binding Motif Is Present in Neuroplastin but Not in Other Synaptogenic CAMs

**FIGURE 1:**
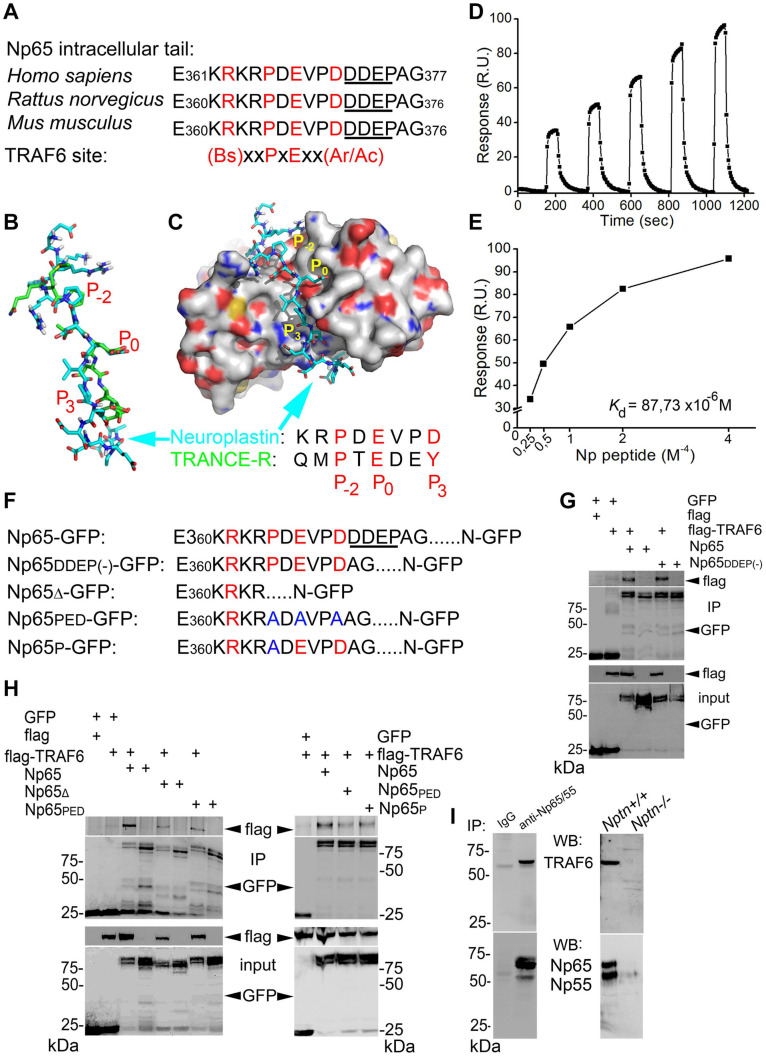
Characterization of the binding of TRAF6 to neuroplastin. **(A)** Potential TRAF6 binding motif in the intracellular tail of neuroplastin 65 (and identical in neuroplastin 55) fits the canonical and specific motif recognized by TRAF6. The alternatively spliced DDEP sequence is underlined. Bs, Ac, and Ar stand for basic, acidic, and aromatic amino acids, respectively. **(B,C)** Neuroplastin-TRAF6 binding in silico. **(B)** Three-dimensional model of the TRAF6 binding motif in the intracellular tail of neuroplastin (cyan) and key amino acids responsible for the binding to TRAF6 fit to the well-known TRAF6 binding motif present in the TRANCE receptor (green). **(C)** Docking of the TRAF6 binding motif of neuroplastin into the TRAF6 C-domain. Similar to the binding of TRANCE receptor to TRAF6 documented by crystallographic data (Yin et al., 2009), interaction of neuroplastin with TRAF6 would be mediated by the Proline (P) in the position P_–2_ Glutamic acid **(E)** in P0, and Aspartic acid **(D)** in P_3_. **(D,E)** Direct binding of the neuroplastin-derived intracellular peptide comprising the TRAF6 binding motif to purified recombinant TRAF6. Time-dependent **(D)** and concentration-dependent **(E)** binding curve for the neuroplastin-TRAF6 binding where obtained using surface plasmon resonance. **(F–H)** Neuroplastin-TRAF6 co-precipitation is drastically decreased by deletion or mutation of key amino acids in the TRAF6 binding motif of neuroplastin. **(F)** Neuroplastin constructs included in the experiments are listed. **(G)** HEK cells were co-transfected with constructs encoding either GFP, Np65-GFP or Np65_DDEP(–)_-GFP and with TRAF6-flag or flag alone for 24 h. Alternatively, **(H)** HEK cells were co-transfected with GFP, Np65-GFP, Np65Δ-GFP (TRAF6 binding motif deficient construct), Np65_PED_-GFP (containing a TRAF6 binding motif with triple substitution to alanine) or Np65_P_-GFP (with single substitution to alanine) and with TRAF6-flag or flag constructs for 24 h. After homogenization, anti-GFP antibody-coupled beads were used to precipitate GFP-tagged complexes. We used anti-Flag or anti-GFP antibodies to detect the proteins as indicated. Representative images from 4-6 independent experiments. **(I)** Three-weeks old rat forebrains (left panel) and hippocampus from 2 weeks-old *Nptn*^+/+^ and *Nptn*^–/–^ mice (right panel) were lysed and homogenized with RIPA lysis buffer and incubated with a KO-controlled antibody recognizing all neuroplastin isoforms raised in rabbit or pre-immune IgG from rabbit for 24 h at 4°C. Precipitated proteins were resolved by SDS-PAGE and immunoblotted with a KO-controlled pan anti-Np65/55 antibody from sheep or an anti-TRAF6 antibody from mouse (see section Materials and Methods).

**FIGURE 2:**
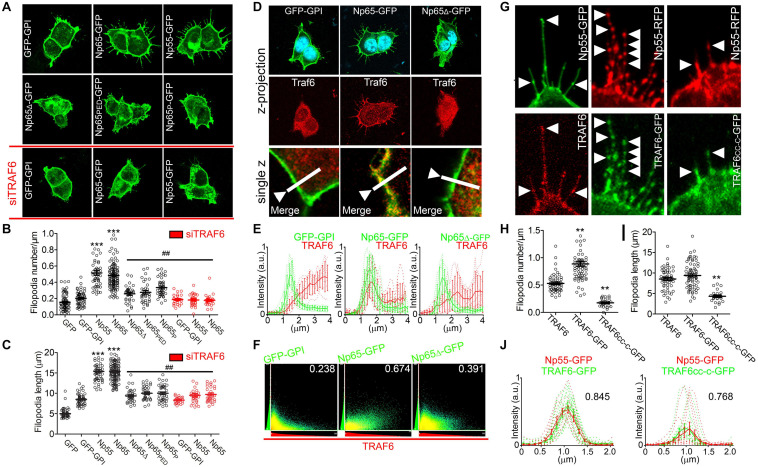
Neuroplastin requires its TRAF6 binding motif and TRAF6 to promote filopodia formation. HEK cells were transfected with plasmids coding for either soluble GFP, membrane-attached GFP-GPI, DDEP insert-containing isoforms Np65-GFP or Np55-GFP, TRAF6 binding motif-deficient Np65Δ-GFP, Np65_PED_-GFP (containing triple substitution to alanine in the TRAF6 binding motif) or Np65_P_-GFP (with single substitution to alanine), full-length TRAF6-GFP or coiled coil-TRAF-C-GFP (TRAF6_cc–c_-GFP). To knockdown endogenous TRAF6, cells were transfected with siRNA against TRAF6 or scrambled siRNA before the transfection with plasmids. After 48 h, cells were fixed with methanol and immunostained with anti-GFP rabbit antibody overnight and with an Alexa-488 secondary antibody. Alternatively, cells were additionally stained with an anti-TRAF6 rabbit antibody followed by a Cy3-conjugated secondary antibody and DAPI. Data and images from *N* = 4–6 independent experiments. **(A–C)** Deletion or mutations of the TRAF6 binding motif of neuroplastin or knock-down of endogenous TRAF6 decrease neuroplastin capacity to promote filopodia. **(A)** Scale bar = 10 μm. **(B)** Filopodia number per micron of plasma membrane (GFP = 0.15 ± 0.01, *n* = 70; GFP-GPI = 0.20 ± 0.01, *n* = 59; Np55-GFP = 0.51 ± 0.02, *n* = 51; Np65-GFP = 0.48 ± 0.01, *n* = 126; Np65Δ-GFP = 0.25 ± 0.01, *n* = 34; Np65_PED_-GFP = 0.27 ± 0.02, *n* = 34; Np65_P_-GFP = 0.33 ± 0.01, *n* = 40; siTRAF6 GFP-GPI = 0.29 ± 0.01, *n* = 30; siTRAF6 Np55-GFP = 0.17 ± 0.01, *n* = 28; siTRAF6 Np65-GFP = 0.18 ± 0.01, *n* = 27) and **(C)** Filopodia length (GFP = 5.07 ± 0.24; GFP GPI = 8.41 ± 0.33; Np55-GFP = 16.68 ± 0.76; Np65-GFP = 16.67 ± 0.48; Np65Δ-GFP = 9.48 ± 0.57; Np65_PED_-GFP = 10.79 ± 0.58; Np65_P_-GFP = 10.78 ± 0.64; siTRAF6 GFP-GPI = 1.73 ± 0.72; siTRAF6 Np55-GFP = 10.18 ± 0.72; siTRAF6 Np65-GFP = 9.77 ± 0.85) were quantified using a semi-automatized Matlab-based algorithm. ***p < 0.001 for the indicated condition vs. GFP and ^##^*p* < 0.01 vs. Np65 using Student‘s *t*-test. **(D**–**F)** Full-length neuroplastin recruits cytosolic TRAF6 to the cell membrane. **(D)** Endogenous TRAF6 is recruited by and co-localizes with Np65-GFP, but not with Np65Δ-GFP nor with GFP-GPI at the plasma membrane. Scale bar = 10 μm. The lower confocal pictures are single z plains and on them, a line scan served to quantify the fluorescence distribution of the GFP-tagged proteins and TRAF6 as shown in **(E)**. **(F)** Co-localization index (Pearson’s coefficient) is displayed for each of the condition as indicated. **(G–J)** Elimination of RING domain abrogates TRAF6 capacity to mediate neuroplastin-promoted filopodia formation. **(G)** The pictures are single z plains acquired by confocal microscopy. Arrow heads point to fluorescent spots formed by Np55-RFP co-localizing with TRAF6-GFP or with TRAF6_cc–c_-GFP. **(H)** Filopodia number (TRAF6 = 0.53 ± 0.02, *n* = 54; TRAF6-GFP = 0.88 ± 0.04, *n* = 69; TRAF6_cc–c_-GFP = 0.17 ± 0.02, *n* = 25) and **(I)** Filopodia length (TRAF6 = 13.44 ± 0.35; TRAF6GFP = 13.89 ± 0.37; TRAF6_cc–c_-GFP = 4.3 ± 0.29) were obtained using a semi-automatized Matlab-based algorithm. **p < 0.01 vs. TRAF6 using Student‘s *t*-test. **(J)** Line scan analysis of TRAF6-GFP- and Np55-RFP-associated fluorescent signals of single spots. Also, the corresponding co-localization index (Pearson’s coefficient) is displayed for each plotting.

Using the ELM database^1^, we identified a single TRAF6 binding motif in the cytoplasmic tail of all neuroplastins from human, rat, and mouse (Figure 1A and Supplementary Figure S1A) matching the well-characterized TRAF6 binding motif (Ye et al., 2002; Sorrentino et al., 2008; Yin et al., 2009). Due to alternative splicing two neuroplastin isoforms Np55 and Np65 differ in an additional Ig domain in the extracellular part, and another alternative splicing event concerns a mini-exon encoding four additional amino acids Asp-Asp-Glu-Pro (DDEP) in the C-terminal part (Langnaese et al., 1997). This DDEP sequence is close to the identified TRAF6 binding motif (Figure 1A). Based on crystallographic studies on the interaction of the TRAF6 TRAF-C domain with the TRANCE receptor (Ye et al., 2002), *in silico* modeling was applied to TRAF6 TRAF-C domain-neuroplastin interaction (Figure 1B and Supplementary Figure S1C). A strikingly similar three-dimensional structure was predicted for the TRAF6 binding motif of neuroplastin when compared to the TRANCE receptor TRAF6 binding motif (Figure 1C and Supplementary Figure S1C). In particular, the coordinates and stereo specificity of key amino acids (Figure 1B, *P*_–2_ = Pro, *P*_0_ = Glu, and *P*_3_ = Aromatic/Acidic) involved in docking of the TRANCE receptor to TRAF6 TRAF-C domain (TRAF-C) were conserved in the TRAF6 binding motif of neuroplastin (Figure 1C and Supplementary Figure S1C). Thus, we conclude that the cytoplasmic tail of neuroplastin displays a proper TRAF6 binding site.

We searched for TRAF6 binding motifs in additional spinogenic CAMs (Supplementary Figure S1B). Rather surprising, the TRAF6 binding motif was not found among other type-1 CAMs known to participate in synapse formation namely *N*-Cadherin (Bozdagi et al., 2010), LRRTM (Linhoff et al., 2009), neuroligins (Varoqueaux et al., 2006), neurexins (Missler et al., 2003), SynCAM1 (Robbins et al., 2010), EphB2 (Henderson et al., 2001), PTPR0 (Jiang et al., 2017) (Supplementary Figure S1B). The data indicate that direct TRAF6 binding is not a generalized feature among spinogenic CAMs, but rather highlight the potential specificity and importance of the association of TRAF6 to neuroplastin.

We sought to confirm that there is a direct physical interaction between TRAF6 and neuroplastin. To this end, we characterized the binding of the purified neuroplastin intracellular peptide containing the TRAF6 binding motif to immobilized recombinant TRAF6 by surface plasmon resonance (Figures 1D,E and Supplementary Figures S1D,E). Binding to TRAF6 was found dependent on neuroplastin peptide concentration, saturable, and displayed a 1:1 stoichiometry. We calculated a *K*_d_ value of 88 μM for the neuroplastin-TRAF6 interaction (Figures 1D,E), which is very similar to the *K*_d_ of 84 μM for the TRANCE receptor-TRAF6 binding (Yin et al., 2009). To establish whether the TRAF6 motif in neuroplastin binds TRAF6 in living cells, we performed co-immunoprecipitation assays from HEK cells transfected with different GFP-tagged constructs of neuroplastins and flag-tagged TRAF6. HEK cells have been successfully used before to evaluate the protein interactions of other spinogenic CAMs at the molecular level (Sarto-Jackson et al., 2012; Jiang et al., 2017). Due to alternative splicing of the primary transcript, both major neuroplastin isoforms Np65 and Np55 can contain the alternative DDEP insert close to their TRAF6 binding motif. To consider potential differences in binding, splicing variants with and without DDEP were tested. DDEP splice variants of Np65-GFP co-precipitated flag-TRAF6 suggesting that the mini exon-encoded insertion is not critical for the binding (Figures 1F,G). Similarly, Np55 with and without DDEP insertion co-precipitated with TRAF6 (Supplementary Figure S1F). In contrast, co-precipitation was largely decreased when GFP-tagged versions of Np65 either with deleted TRAF6 binding motif (Np65Δ-GFP) or with triple (Np65_PED_-GFP) or single (Np65_P_-GFP) amino acid substitutions in the binding motif were used (Figures 1F,G) as confirmed by densitometric analysis (Supplementary Figure S1G). Additionally, pull-down assays demonstrated that Np65-GFP isolated from HEK cells binds similarly well to purified recombinant GST-TRAF6 or to the GST-TRAF6 C-domain (coiled coil + TRAF-C domain, GST-TRAF6_cc–c_) (Supplementary Figures S1D,E). The data support the idea that the TRAF6 binding motif in the cytoplasmic tail of neuroplastin is fully capable of binding the TRAF-C domain of TRAF6. Using highly specific neuroplastin antibodies (Herrera-Molina et al., 2014; Bhattacharya et al., 2017; Korthals et al., 2017), we could also show that TRAF6 co-immunoprecipitated with neuroplastin isoforms from brain extracts of 3 weeks-old rats or 2 weeks-old *Nptn*^+/+^ mice, but not from 2 weeks-old *Nptn*^–/–^ mice (Figure 1I).

### TRAF6 Mediates the Formation of Filopodial Structures by Neuroplastin

We have reported disorganization of polymerized actin in dendrites of *Nptn*^–/–^ primary hippocampal neurons (Herrera-Molina et al., 2014). Coincidently, TRAF6 is known to increase of actin polymerization (Armstrong et al., 2002; Wang et al., 2006; Yamashita et al., 2008). Therefore, we performed experiments to explore if and how TRAF6 and neuroplastin interact to increase actin-based filopodia formation in HEK cells. Over-expression of either of the two neuroplastin isoforms Np55 and Np65 in HEK cells was sufficient to induce a massive increase in filopodia number and length as compared to control cells transfected with either soluble or membrane-attached GFP (Figures 2A–C). DDEP-lacking variants of Np55 or Np65 were as effective as the ones that carry the insert to promote filopodial structures (Supplementary Figures S2A–D). However, the capacity of neuroplastin to promote filopodia was abolished by mutation or elimination of the TRAF6 binding site (i.e., Np65Δ-GFP, Np65_PED_-GFP, Np65_P_-GFP) (Figures 2A–C). Furthermore, after decreasing protein levels of endogenous TRAF6 by ∼80% using a specific siRNA (Supplementary Figures S2E,F), neither expression of Np65-GFP nor of Np55-GFP did increase the number or length of filopodia in HEK cells (Figures 2A–C). Thus, Np55 and Np65 (±DDEP) are similarly effective to promote the formation of filopodial structures and seem to require endogenous TRAF6 and binding to their TRAF6 motifs to do so.

TRAF6 translocates from the cytoplasm to the membrane by recruitment to integral membrane proteins with TRAF6 binding domains (Yin et al., 2009; Wu, 2013). Therefore, we tested whether neuroplastins via their C-terminal TRAF6 binding motif have the capacity to recruit endogenous TRAF6 to the plasma membrane. In HEK cells transfected with GPI-anchored GFP or with Np65Δ-GFP, TRAF6 immunoreactivity was primarily located in the cytoplasm (Figure 2D). In contrast, TRAF6 immunoreactivity was abundantly associated with the plasma membrane in cells expressing recombinant Np65-GFP (Figure 2D) or other variants of neuroplastin (Supplementary Figure S2A). Analyses of co-distribution (Figure 2E) and co-localization (Figure 2F) confirmed that plasma membrane-associated TRAF6 co-localizes with Np65. Thus, neuroplastin recruits TRAF6 to the plasma membrane and thereby changes its subcellular localization. This capacity is independent of the presence or absence of the DDEP insert. These experiments favorably complement the binding assays in co-transfected HEK cells (Figure 1).

Next, we asked whether the recruitment and binding of TRAF6 by neuroplastin mediate filopodia formation. To test this prediction, we co-expressed GFP-tagged TRAF6 (TRAF6-GFP) with Np55-RFP. Clearly, co-expression of TRAF6-GFP fostered the increase of filopodia number by Np55-GFP (Figures 2G–I). Intriguingly, endogenous TRAF6 and TRAF6-GFP co-localized with Np55-RFP in filopodia-associated microscopic spots (Figure 2G). Indeed, analyses of fluorescent intensity and distribution revealed high co-localization of TRAF6-GFP with Np55-RFP in single spots of filopodia (Figure 2J). The potential involvement of the N-terminal RING domain of TRAF6 was tested using TRAF6_cc–c_-GFP containing the coiled coil and TRAF-C domains and lacking the N-terminal domain (Supplementary Figures S1D,E). Despite being recruited to the plasma membrane and co-localized with Np55-RFP (Figures 2G,J), TRAF6_cc–c_-GFP blocked neuroplastin-induced filopodia formation (Figures 2G–J). Accordingly, the recruitment and binding of TRAF6_cc–c_ by neuroplastin is insufficient to promote filopodial structures. Because the RING domain is well-known to be responsible for three-dimensional assembly of functional TRAF6 lattice-like structures (Yin et al., 2009; Ferrao et al., 2012; Wu, 2013), we conclude that only the recruitment and binding of fully functional TRAF6 increases formation of filopodial structures by neuroplastin.

### Neuroplastin Promotes the Formation of Spinogenic Dendritic Protrusions

**FIGURE 3:**
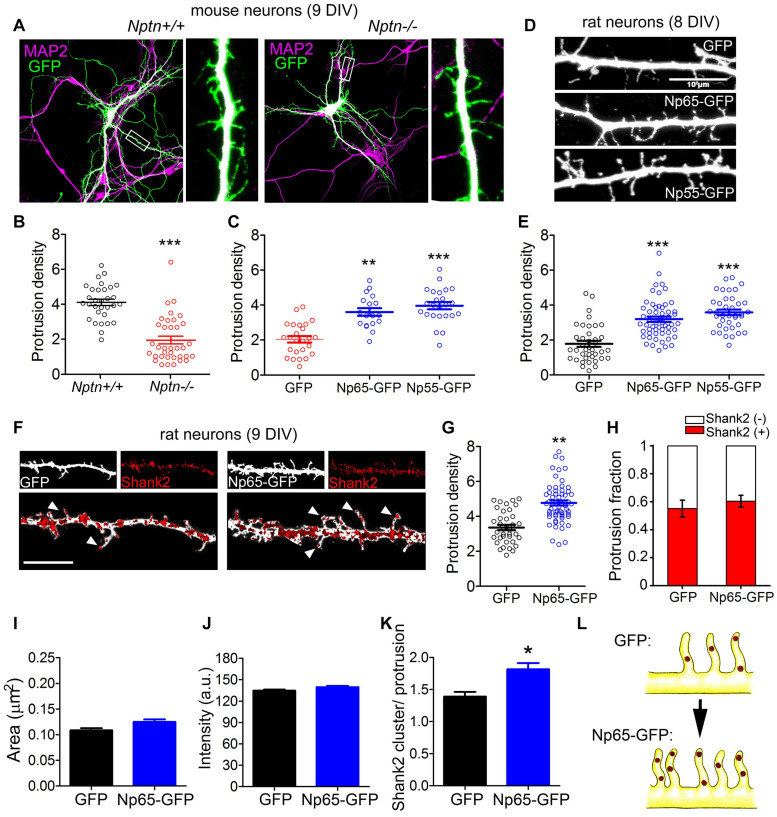
Neuroplastin promotes formation of dendritic protrusions. **(A–C)** Reduced number of dendritic protrusions in *Nptn*^–/–^ compared to *Nptn*^+/+^ mouse primary hippocampal neurons at 9 DIV. **(A)** *Nptn*^–/–^ and *Nptn*^+/+^ neurons transfected with GFP-encoding plasmids at 6–7 DIV using Lipofectamine. At 9 DIV, neurons were fixed and stained with anti-GFP antibody followed by an Alexa 488-conjugated antibody to enhance their intrinsic fluorescence (green) and with anti-MAP2 antibodies followed by a proper secondary antibody to detect dendrites (magenta). Images were obtained using a confocal microscope. Scale bar = 100 μm. **(B)** Protrusion density (number of dendritic protrusions per 10 μm) of GFP-filled *Nptn*^–/–^ and *Nptn*^+/+^ neurons (circles) is expressed as mean ± SEM from three independent cultures. ***p < 0.001 between genotypes using Student‘s *t*-test (*Nptn*^+/+^ GFP = 4.12 ± 0.18, *n* = 33; *Nptn*^–/–^ GFP = 1.72 ± 0.19, *n* = 36). **(C)** Protrusion density of GFP-, Np65- GFP-, or Np55-GFP-expressing *Nptn*^–/–^ neurons from two independent cultures. ***p < 0.001 or **p < 0.01 vs. *Nptn*^–/–^ GFP using Student‘s t-test (*Nptn*^–/–^ GFP = 1.92 ± 0.22, *n* = 26; *Nptn*^–/–^ Np65-GFP = 3.67 ± 0.18, *n* = 20; *Nptn*^–/–^ Np55-GFP = 3.77 ± 0.19, *n* = 26). **(D,E)** Both neuroplastin isoforms increase dendritic protrusion density in rat neurons at 8 DIV. **(D)** Confocal images show rat neurons transfected with plasmids encoding GFP, Np65-GFP or Np55-GFP at 7 DIV. At 8 DIV, neurons were fixed and stained with anti-GFP antibody followed by an Alexa 488-conjugated antibody (white). Scale bar = 10 μm **(E)** Protrusion densities of 40–50 neurons per group (circles) from 3 to 4 independent cultures. ***p < 0.001 vs. GFP transfected cells using Student‘s *t*-test (GFP = 1.95 ± 0.19, *n* = 39; Np65-GFP = 3.23 ± 0.14, *n* = 56; Np55-GFP = 3.58 ± 0.16, *n* = 38). **(F–H)** Overexpression of Np65-GFP increases the number of newly formed Shank2-containing dendritic protrusions. **(F)** Cropped confocal images of dendritic segments of rat neurons transfected with GFP or Np65-GFP at 7 DIV. At 9 DIV, neurons were fixed and stained with primary antibodies against GFP (white) and Shank2 (red). Arrow heads point to Shank2 spots in dendritic protrusions. Scale bar = 10 μm. **(G)** Protrusion density (GFP = 3.151 ± 0.182, *n* = 48; Np65-GFP = 4.642 ± 0.145, *n* = 54) and **(H)** Distribution of Shank2-positive and Shank2-negative protrusions were calculated as a fraction from *n* = 40–50 neurons per group from *N* = 3 independent experiments. Plots display mean ± SEM as indicated. **p < 0.01 for Np65-GFP vs. GFP using Student‘s *t*-test [Shank2(+): GFP = 0.54 ± 0.07; Np65-GFP = 0.60 ± 0.06]. **(I–K) (I)** Size of puncta (area; GFP = 0.10 ± 0.01, *n* = 747; Np65-GFP = 0.11 ± 0.01, *n* = 738), **(J**) Fluorescence intensity (GFP = 127.6 ± 2.1; Np65-GFP = 131.5 ± 1.9) and **(K)** Number of Shank2 clusters/protrusion in neurons (GFP = 1.46 ± 0.17, *n* = 43; Np65-GFP = 1.91 ± 0.18, *n* = 49) of the experiments displayed in **(F)**. *p < 0.05 between Np65-GFP-expressing and GFP-expressing neurons using Student‘s *t*-test. **(L)** The upper sketch on the left illustrates dendritic protrusions enriched on Shank2 in control GFP-filled hippocampal neurons at 9 DIV. Np65-GFP-expressing neurons (lower sketch) display more spinogenic protrusions with Shank2 clusters.

Neuroplastin has been related to synapse formation *in vitro* and *in vivo* (Herrera-Molina et al., 2014; Amuti et al., 2016; Carrott et al., 2016; Zeng et al., 2016), but the underlying molecular mechanism is unknown. As TRAF6 mediated filopodia formation by neuroplastin in HEK cells, we studied the involvement of the two proteins in the formation of dendritic protrusions, which act as precursors of spines in mature neurons (Ziv and Smith, 1996; McClelland et al., 2010). By confocal microscopy we quantified the number of protrusions per 10 μm length expanding from MAP2-stained dendrites of GFP-filled pyramidal neurons in primary hippocampal cultures from wild-type and neuroplastin-(*Nptn*-)deficient mice (Figures 3A,B). Absence of neuroplastin gene expression resulted in reduced density of dendritic protrusions in *Nptn*^–/–^ compared to *Nptn*^+/+^ hippocampal neurons at 9 days *in vitro* (DIV). This phenotype was rescued by transfection of mutant neurons with recombinant neuroplastin isoforms Np55-GFP or Np65-GFP at 9 DIV (Figure 3C). In parallel experiments with rat primary hippocampal neurons, we observed that the over-expression of either neuroplastin isoform promotes dendritic protrusion density significantly (Figures 3D,E). Rat neurons transfected with either Np55-GFP or Np65-GFP at 7 DIV displayed higher density of dendritic protrusion than control GPF-transfected neurons when evaluated at 8 DIV (Figures 3D,E).

To characterize the spinogenic nature of neuroplastin-promoted dendritic protrusions, we evaluated the presence of Shank2, a key organizer of excitatory postsynaptic structures (Roussignol et al., 2005) and well-established marker for excitatory spines (Grabrucker et al., 2011; Sarowar and Grabrucker, 2016). While transfection of Np65-GFP at 7 DIV increased dendritic protrusion density compared to control GFP, the relative abundance of Shank2-positive vs. Shank2-negative protrusions (Protrusion fraction) was not different between Np65-GFP-tranfected and GFP-transfected rat neurons at 9 DIV (Figures 3F–H). Although area and intensity of Shank2 clusters were not different between Np65-GFP- and GFP-expressing dendrites at 9 DIV (Figures 3I,J), the number of Shank2 clusters per dendritic protrusion was higher in Np65-GFP- vs. GFP-expressing dendrites at 9 DIV (Figure 3K). Thus, Np65-GFP-overexpressing neurons display an increased number of newly formed postsynapses defined as dendritic protrusions containing a higher number of Shank2 clusters compared to GPF-filled control neurons (Figure 3L). These results support the idea that neuroplastin raises the density of spinogenic protrusions during the development of neurons.

### Neuroplastin Promotes Dendritic Protrusions in a Restricted Developmental Time Period and Requires TRAF6

**FIGURE 4:**
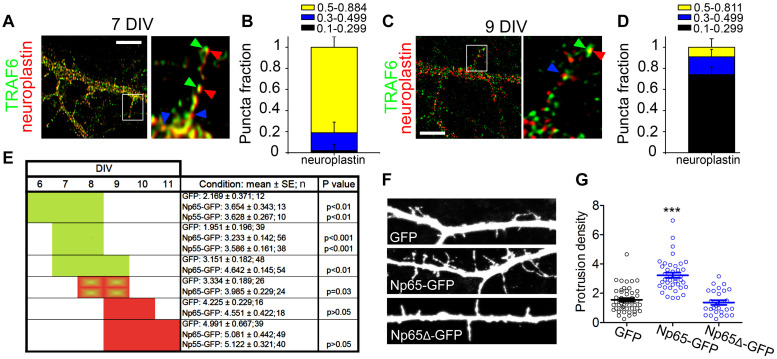
Permissive time period for protrusion induction by neuroplastin and TRAF6. **(A,D)** To avoid interference with the intracellular interaction of TRAF6 with the tail of neuroplastin, methanol-fixed rat hippocampal neurons at 7 and 9 DIV were stained with a sheep pan-neuroplastin antibody recognizing the common extracellular Ig2-like domain of Np55 and Np65 and with an rabbit anti-TRAF6 antibody recognizing the amino acids 1–274 located at the intracellular N-terminal of TRAF6. These primary antibodies were followed by proper fluorophore-tagged secondary antibodies, mounted, and imaged using a 100x objective and confocal microscopy. Images were deconvolved to eliminate optic aberrations and improve resolution (see section “Materials and Methods”). **(A,C)** Confocal pictures and digital magnifications of dendritic protrusions are shown. Green arrows show co-localized TRAF6 spots, red arrows point to co-localized neuroplastin spots, and blue arrows show neuroplastin spots with lower co-localization degree. Scale bars = 5 μm. **(B,D)** Quantification of the fractions of neuroplastin spots co-localized with TRAF6 spots was performed using single z-planes. The degree of co-localization of neuroplastin was scored as high (Pearson’s coefficient from 0.5 to 0.884 or 0.811), medium (0.3–0.499) or low/no-localization (0.1–0.299). 51 **(B)** or 81 **(D)** dendritic protrusions from 2 independent cultures were analyzed. **(E)** Neuroplastin promotes dendritic protrusion during a distinct time window in the development. The panel summarizes the results from transfections of Np55-GFP or Np65-GFP at 6, 7, 8, and 9 DIV as indicated. After 24 or 48 h, evaluation of dendritic protrusion density was performed at the end of each experimental series. In green, time periods when transfections effectively promoted protrusion density. In red, neuroplastin did not promote protrusion density. Data from 3 independent cultures. **(F,G)** Np65 requires its TRAF6 binding motif to foster formation of dendritic protrusions. **(F)** Dendritic segments of 8 DIV rat neurons expressing the indicated proteins upon transfection are shown. **(G)** Protrusion densities from three independent cultures are expressed as the mean ± SEM (GFP = 1.72 ± 0.15, *n* = 52, Np65-GFP = 3.63 ± 0.11, *n* = 43; Np65Δ-GFP = 1.64 ± 0.16, *n* = 28). ***p < 0.001 vs. GFP using Student‘s *t*-test. Images and data from 3 independent cultures.

We tested whether and when neuroplastin requires TRAF6 to raise dendritic protrusion density in neurons. Using confocal microscopy and image deconvolution procedures in single z-planes, we assessed the co-localization/co-distribution of endogenous TRAF6 and endogenous neuroplastin in young neurons at 7 and 9 DIV (Figures 4A–D). At 7DIV, ∼95% of neuroplastin spots displayed high or medium degrees co-localization with TRAF6 spots (Figures 4A,B) indicating that both proteins are in close proximity and may interact in dendritic protrusions during this earlier stage of neuronal development. The degree of neuroplastin-TRAF6 co-localization was lower at 9 DIV as only ∼15% of neuroplastin spots showed some co-localization with TRAF6 (Figures 4C,D). Then, we evaluated further the timing for neuroplastin-mediated increase in dendritic protrusions by transfecting rat hippocampal neurons with either Np55-GFP or Np65-GFP at DIV 6, 7, 8, or 9. The density of dendritic protrusions was evaluated 24 or 48 h after transfection (Figure 4E). Neurons transfected with either Np55-GFP or Np65-GFP at 6 or 7 DIV displayed higher density of dendritic protrusion than control GPF-transfected neurons when evaluated at 8 or 9 DIV (green blocks, Figure 4E). Later transfections of Np65- or Np55-GFP performed at 9 DIV were ineffective to raise the protrusion density in rat neurons analyzed at 10 or 11 DIV (red blocks, Figure 4E). Therefore, we can conclude that neuroplastin increases the density of dendritic protrusions during a time period of major synapse formation in neuronal development. Following this observation, we elucidate if neuroplastin requires its intracellular TRAF6 binding site to promote dendritic protrusions at 8 DIV. While Np65-GFP fostered the density of dendritic protrusions as expected, Np65Δ-GFP failed to do so (Figures 4F,G).

### TRAF6 Confers the Spinogenesis-Promoting Capacity to Neuroplastin

**FIGURE 5:**
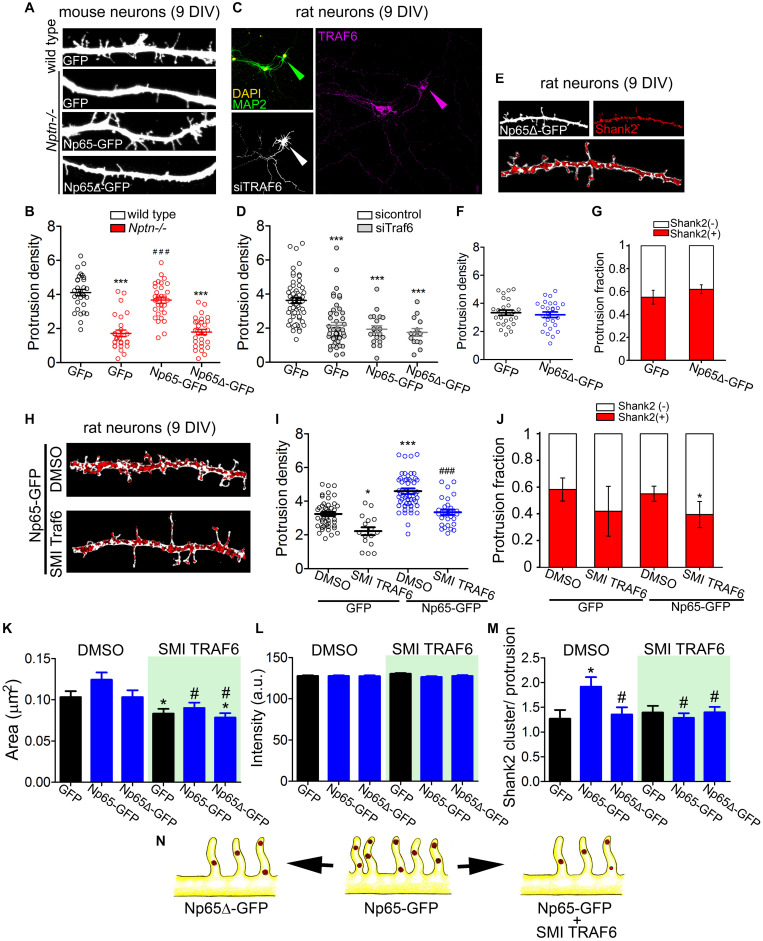
TRAF6 mediates dendritic protrusion formation via neuroplastin. **(A,B)** The TRAF6 binding motif-deficient Np65Δ-GFP does not rescue dendritic protrusion formation in *Nptn*^–/–^ neurons. **(A)** Confocal images of segments of dendrites of *Nptn*^+/+^ and *Nptn*^–/–^ neurons transfected with plasmids encoding GFP, Np65-GFP or Np65Δ-GFP at 7 DIV. At 9 DIV, these neurons were fixed and stained with anti-GFP antibodies followed by an Alexa 488-conjugated antibody (green). **(B)** Protrusion densities from 2 independent cultures were used to obtain the mean ± SEM as indicated (*Nptn*^+/+^ GFP = 4.12 ± 0.18, *n* = 34; *Nptn*^–/–^ GFP = 1.72 ± 0.19, *n* = 27; *Nptn*^–/–^ Np65-GFP = 3.67 ± 0.18, *n* = 33; *Nptn*^–/–^ Np65Δ-GFP = 1.79 ± 0.16, *n* = 33). ***p < 0.001 vs. GFP-filled wild type neurons and ^###^*p* < 0.001 vs. GFP-filled *Nptn*^–/–^ neurons using Student‘s *t*-test. **(C,D)** TRAF6 knockdown prevents the increase of dendritic protrusions induced by Np65-GFP in hippocampal neurons. Neurons were co-transfected with either control scrambled siRNA or siRNA against TRAF6 mRNA and with GFP-encoding plasmid (6 DIV). Additionally, neurons were co-transfected with siRNA and Np65-GFP or Np65Δ-GFP. After 72 h, neurons were stained with anti-MAP2 and anti-TRAF6 antibodies to control neuronal morphology and TRAF6 KD, respectively. Only neurons with ≥60% reduction in TRAF6 immunoreactivity (arrow heads in **C**) were considered for the counting of dendritic protrusions. **(D)** Transfected neurons from 4 independent cultures were analyzed (sicontrol GFP = 3.73 ± 0.16, *n* = 59; siTRAF6 GFP = 2.16 ± 0.18, *n* = 49; siTRAF6 Np65-GFP = 2.09 ± 0.16, *n* = 22; siTRAF6 Np65Δ-GFP = 1.69 ± 0.17, *n* = 14). ***p < 0.001 vs. sicontrol GFP using Student‘s *t*-test. Scale bar = 100 μm. **(E–G)** Expression of Np65Δ-GFP does not increase the number of dendritic protrusions in 9 DIV-old rat hippocampal neurons. **(E)** Dendritic segments of neurons expressing GFP or Np65Δ-GFP and stained with antibodies against GFP (white) and Shank2 (red clusters) were photographed using confocal microscopy. Images were processed to identify Shank2 clusters of interest (see section “Materials and Methods”). Scale bar = 10 μm. **(F)** Quantification of the protrusion densities and **(G)** the distribution of Shank2-positive and Shank2-negative protrusions from 20-30 neurons per group from 3 independent cultures [Shank2(+): GFP = 0.55 ± 0.06; Np65Δ-GFP = 0.62 ± 0.04]. **(H–J)** TRAF6 inhibition decreases formation of dendritic protrusions. **(H)** 7 DIV-old rat neurons were transfected with Np65-GFP, treated with the TRAF6 inhibitor SMI 6860766 (SMI TRAF6, 2 μm) for 48 h, fixed, and stained for GFP (white) and Shank2 (red clusters) at 9 DIV. Scale bar = 10 μm. **(I)** Protrusion density (DMSO GFP = 3.24 ± 0.118, *n* = 47; SMI TRAF6 GFP = 2.22 ± 0.23, *n* = 16; DMSO Np65-GFP = 4.59 ± 0.16, *n* = 56; SMI TRAF6 Np65-GFP = 3.34 ± 0.16, *n* = 28) and **(J)** Distribution of Shank2-positve and Shank2-negative protrusions from transfected neurons per group from 3 independent cultures are displayed. *p < 0.05 or ***p < 0.001 vs. DMSO GFP and ^###^*p* < 0.001 vs. SMI TRAF6 GFP using Student’s *t*-test [Shank2(+): GFP DMSO = 0.58 ± 0.08; SMI TRAF6 = 0.52 ± 0.19; Np65-GFP DMSO = 0.55 ± 0.06; Np65-GFP SMI TRAF6 = 0.42 ± 0.09]. **(K–M)** From the experiments in **(H–J)** we calculated **(K)** the area of Shank2 clusters (DMSO GFP = 0.105 ± 0.004; DMSO Np65-GFP = 0.137 ± 0.004; DMSO Np65Δ-GFP = 0.106 ± 0.003; SMI TRAF6 GFP = 0.093 ± 0.003; SMI TRAF6 Np65-GFP = 0.094 ± 0.006; SMI TRAF6 Np65Δ-GFP = 0.098 ± 0.005), **(L)** the fluorescence intensity of the clusters (DMSO GFP = 134.6 ± 1.4; DMSO Np65-GFP = 139.5 ± 1.9; DMSO Np65Δ-GFP = 138.4 ± 2.1; SMI TRAF6 GFP = 133.0 ± 1.7; SMI TRAF6 Np65-GFP = 134.0 ± 1.8; SMI TRAF6 Np65Δ-GFP = 134.3 ± 1.6), and **(M)** the number of Shank2 clusters per protrusion (DMSO GFP = 1.36 ± 0.17; DMSO Np65-GFP = 1.92 ± 0.14; DMSO Np65Δ-GFP = 1.35 ± 0.14; SMI TRAF6 GFP = 1.39 ± 0.13; SMI TRAF6 Np65-GFP = 1.28 ± 0.09; SMI TRAF6 Np65Δ-GFP = 1.40 ± 0.10). *p < 0.05 between Np65-GFP-expressing and GFP-expressing neurons using Student’s *t*-test. #*p* < 0.05 between the treatments for the same transfection. **(N)** Neuroplastin requires both its TRAF6 binding motif and endogenous TRAF6 activity to promote spinogenic protrusion density. The illustration in the middle shows Np65-GFP-expressing neurons with increased density of Shank2-containing spinogenic protrusions. This phenotype is no longer observed when the TRAF6 binding motif is deleted from the Np65 intracellular tail (Np65Δ-GFP, left). TRAF6 blockage decreases both the density of protrusions and fraction of protrusions with Shank2 clusters (right).

We evaluated whether TRAF6 is essential for neuroplastin to raise the density of dendritic protrusions during the defined critical time period of neuronal development. Consistently, the protrusion density in *Nptn*^–/–^ dendrites expressing Np65-GFP was higher than in control *Nptn*^–/–^ dendrites expressing GFP at 9 DIV (Figures 5A,B). Np65Δ-GFP failed to rescue the dendritic protrusion density in *Nptn*^–/–^ neurons (Figures 5A,B). We also confirmed the specificity of the TRAF6-neuroplastin interaction to increase the density of dendritic protrusions in rat neurons co-transfected with TRAF6-specific siRNA (characterized in Supplementary Figures S2E,F) and with GFP-, Np65-GFP or Np65Δ-GFP at 6 DIV. When TRAF6 levels were knocked down by 60% or more at 9 DIV, the dendritic protrusion density was reduced in GFP-, Np65- GFP-, and Np65Δ-GFP-expressing neurons (Figures 5C,D). Additionally, we evaluated whether Np65Δ-GFP affects the number of protrusions and interferes with the normal enrichment of Shank2 in dendritic protrusions in rat neurons at 9 DIV. The density of dendritic protrusions and the distribution of Shank2-positive vs. Shank2-negative protrusions were similar between GFP- and Np65Δ-GFP-expressing rat neurons at 9DIV (Figures 5E–G). These data show that, in contrast to Np65-GFP, Np65Δ-GFP neither rescued impaired spinogenesis in *Nptn*^–/–^ neurons nor increased the number of dendritic protrusions in rat neurons. Independently of the rodent model from which neurons were derived, Np65 depends on its TRAF6 motif and TRAF6 expression to increase the density of spinogenic protrusions in hippocampal neurons.

To confirm further that endogenous TRAF6 is involved in neuroplastin-mediated dendritic protrusion formation, we used a small molecule inhibitor 6860766 (SMI TRAF6), which reversibly binds the TRAF-C domain of TRAF6 blocking its capacity to interact with its binding partners (Chatzigeorgiou et al., 2014; van den Berg et al., 2015), in rat hippocampal neurons at 9 DIV. SMI TRAF6 (2 μM) reduced the density of protrusions in GFP- and in Np65-GFP-expressing neurons compared to vehicle-treatment (0.01% DMSO) (Figures 5H,I). Treatment with SMI TRAF6 decreased the fraction of Shank2-positive protrusions in Np65-GFP-expressing neurons slightly but significantly (Figure 5J). SMI TRAF6 also decreased the area, but not the intensity of Shank2 clusters, and it reduced the number of Shank2 clusters per protrusion in Np65-GFP-expressing neurons to the level of controls (Figures 5K–M). Moreover, SMI TRAF6 treatment evidenced that the size of Shank2 clusters depends on TRAF6 (Figure 5K). Thus, neuroplastin strictly requires its TRAF6 binding motif and TRAF6 expression to increase dendritic protrusion density in hippocampal neurons. Either deficiency of these pre-requisites abrogates the spinogenic capacity of neuroplastin (Figure 5N).

Neuroplastin interacts through its transmembrane domain (Schmidt et al., 2017; Gong et al., 2018) with all four plasma membrane Ca^2+^ ATPases (PMCA1-4) in mature neurons (Herrera-Molina et al., 2017) and immune cells (Korthals et al., 2017). Thus, we addressed the question whether neuroplastin requires PMCA to promote dendritic protrusion density. Consistent with our previous report (Herrera-Molina et al., 2017), Np65-GFP and Np65Δ-GFP were similarly effective to increase protein levels of PMCA2 compared to GFP when co-transfected in HEK cells (Supplementary Figures S3A,B). In rat hippocampal neurons at 9 DIV, confocal microscopy revealed that Np65-GFP and Np65Δ-GFP were effective to increase endogenous PMCA protein levels (Supplementary Figures S3C,D). Although PMCA inhibition seemed to slightly enlarge protrusions, the density of protrusions was not affected in GFP-filled (Supplementary Figures S3E,F) nor in Np65-GFP-expressing neurons at 9 DIV (not shown). Thus, the spinogenic function of neuroplastin is not critically dependent on PMCA levels or activity.

### TRAF6 Effect on Synaptogenesis Impacts Neuronal Activity

**FIGURE 6:**
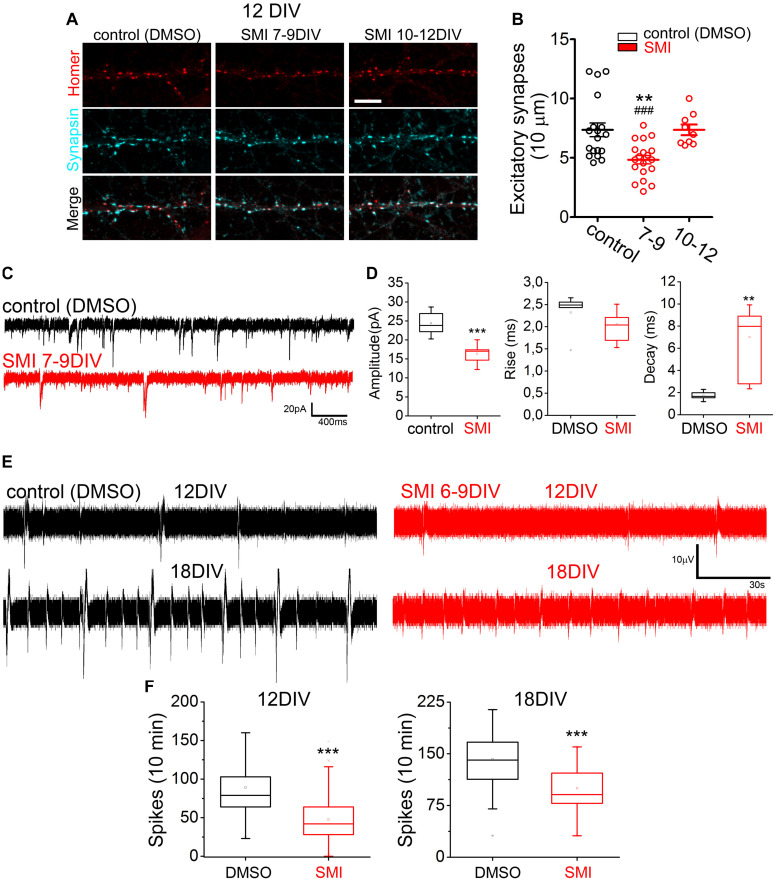
TRAF6 blockage during neuronal development reduces synapse formation affecting neuronal activity. **(A**,**B)** Treatment with SMI TRAF6 reduces the number of excitatory synapses. **(A)** Representative confocal images of dendritic segments stained with antibodies against synaptic markers (red, postsynaptic Homer; cyan, presynaptic Synapsin-1) at 12 DIV. As indicated, rat hippocampal neurons were previously treated with SMI TRAF6 or with the solvent only for 48 h between days 7–9 or 10–12. Scale bar = 10 μm. **(B)** Quantification of the number excitatory synapses per 10 μm of dendritic segment from *N* = 3 independent cultures (control = 7.36 ± 0.58, *n* = 19; 7–9 = 4.84 ± 0.35, *n* = 19; 10–12 = 7.36 ± 0.45, *n* = 9). **p < 0.01 vs. control and ^###^*p* < 0.001 vs. 10–12 using Student’s *t*-test. **(C,D)** Treatment with SMI TRAF6 impairs mEPSCs in hippocampal neurons. **(C)** Representative traces of intracellular recordings of mEPSCs. **(D)** Quantification of the amplitude (DMSO = 24.389 ± 1.531; SMI = 16.384 ± 0.829), rise time (DMSO = 2.320 ± 0.2150; SMI = 2.054 ± 0.113), and decay time (DMSO = 1.729 ± 0.190; SMI = 7.008 ± 1.039) of mEPSCs of 12 DMSO-treated and 14 SMI TRAF6-treated neurons from *N* = 4 independent cultures. ***p < 0.001 or **p < 0.01 vs. DMSO using Student’s *t*-test. **(E,F)** Reduced network activity in SMI TRAF6-treated hippocampal neurons grown on MEAs. **(E)** Traces of extracellularly recorded neuronal activity obtained consecutively when neurons were 12 and also 18 DIV. **(F)** Quantification of the number of spikes per electrode were obtained using Matlab (12 DIV: DMSO = 89.2 ± 3.5, SMI = 47.6 ± 1.9; 18 DIV: DMSO = 142.5 ± 2.8, SMI = 79.5 ± 2.2) from *N* = 3 or 4 independent cultures.

To evaluate long-term implications of TRAF6 blockage during the critical time window when neuroplastin required this factor to foster spinogenesis (Figure 4), we treated young rat hippocampal neurons with SMI TRAF6 (2 μM) or with vehicle (0.01% DMSO) during various time periods and then analyzed the number of excitatory synapses (homer-positive puncta matching synapsin-positive puncta, Herrera-Molina et al., 2014) per 10 μm dendrite (Figures 6A,B). Treatment with SMI TRAF6 from 7 to 9 DIV was sufficient to significantly reduce the number of excitatory synapses at 12 DIV. In contrast, neurons treated with SMI TRAF6 from 10 to 12 DIV displayed a similar number of synapses than the vehicle-treated neurons at 12 DIV (Figures 6A,B). These data confirm that TRAF6 plays a critical developmental role in the formation of ∼25% of hippocampal excitatory synapses *in vitro*.

TRAF6 blockage slightly affected some characteristics of excitatory synapses formed in the absence of TRAF6 function. Evaluation of the area and fluorescence intensity of homer- and synapsin-positive puncta showed that the treatment with SMI TRAF6 from 7 to 9 DIV, but not from 10 to 12 DIV resulted only in a minor change in the area of postsynaptic homer-positive puncta of the synapses (Supplementary Figures S4A,B). On the other hand, area and fluorescence intensity of presynaptic synapsin-positive puncta were in all cases unaltered (Supplementary Figures S4A,B) indicating that synapses formed in the presence of SMI TRAF6 display an almost normal expression and distribution of the synaptic markers. Then, we tested whether the activity of the formed synapses is altered by TRAF6 blockade. Presynaptic uptake of synaptotagmin-1 antibody – reporting vesicle release and recycling driven by intrinsic network activity – showed a slightly decreased activity in mature excitatory (VGAT-negative) and inhibitory (VGAT-positive) presynapses after treatment with SMI TRAF6 from 7 to 9 DIV, but not from 10 to 12 DIV (Supplementary Figure S4C,D). To interpret the physiological significance of these results, we calculated the area of vesicular release (mean area of puncta) and the activity level (mean intensity per pixel) for each presynapse type. From these data (Supplementary Figure S4E), we conclude that inhibitory synapses formed rather normally in the presence of SMI TRAF6 and that its decreased activity results from adaptation to reduced formation of spinogenic dendritic protrusions (Figures 4, 5) resulting in a lower density of excitatory synapses (Figures 6A,B).

To confirm that reduced synaptogenesis by TRAF6 blockage impacts synaptic transmission of matured neurons, primary hippocampal neurons were treated with SMI TRAF6 or vehicle from 6 to 9 DIV, let to mature, and impaled to record intracellularly miniature excitatory postsynaptic currents (mEPSCs) using patch-clamp technique in the presence of 1 μM TTX at 18–23 DIV (Figure 6C). In SMI TRAF6-treated neurons, both amplitude and decay time of mEPSCs were altered, whereas rise time remained practically unchanged compared to vehicle-treated neurons (Figure 6D) indicating physiological alterations at the postsynaptic levels. To confirm further the functional relevance of our findings for neuronal physiology, we evaluated the effect of TRAF6 blockade on network-driven activity of hippocampal neurons cultured on multi-electrode arrays (Figure 6E). Consistent with cell biological and electrophysiological evidence described above, neurons treated with SMI TRAF6 from 6 to 9 DIV displayed lower numbers of extracellular spikes at 12 and 18 DIV, as compared to neurons in control arrays (Figure 6F). This long-lasting impact on neuronal activity highlights the relevance of TRAF6 signaling in spinogenesis during a particular time window of the neuronal development, i.e., 6 to 9 DIV.

## Discussion

Our study addresses the question of how timely orchestrated signaling mechanisms allow neurons to form synapses to communicate with each other. Here, we identified a specific signaling mechanism that, during a critical time window in the neuronal development in primary neuronal cultures, regulates the capacity of neurons to form an adequate density of excitatory synapses. In particular, our findings not only uncover a novel function for TRAF6 in neuronal development but also link it to neuroplastin – shown to be relevant *in vivo* for defining numbers of excitatory synapses and balancing excitation and inhibition in the brain.

### TRAF6-Neuroplastin Binding and Spinogenic Cell Signaling

An important finding is that neuroplastin harbors a single intracellular binding motif to bind TRAF6. The intracellular sequence RKRPDEVPD of neuroplastin fulfilled structural and three-dimensional criteria as well as binding affinity to be a proper TRAF6 binding motif (Ye et al., 2002; Sorrentino et al., 2008; Yin et al., 2009). TRAF6 was only effectively co-precipitated by neuroplastin with an intact TRAF6 binding motif regardless of the presence or absence of the mini-exon-encoded DDEP insert. Not surprisingly (Schultheiss et al., 2001; Yin et al., 2009; Ferrao et al., 2012; Wu, 2013), endogenous TRAF6 and GFP-tagged TRAF6 were recruited into the regularly spaced cell membrane-associated puncta by neuroplastin only when the TRAF6 binding motif was intact. Elimination of the lattice-forming RING domain did not prevent TRAF6 recruitment by neuroplastin but abrogated the capacity of the transmembrane glycoprotein to promote the formation of filopodial structures. After translocation from the cytosol, TRAF6 forms micrometric and geometrically organized lattice-like supramolecular structures that host downstream cell signaling elements beneath the cell membrane (Schultheiss et al., 2001; Yin et al., 2009; Ferrao et al., 2012; Wu, 2013). Thus, it is realistic to conclude that upon TRAF6 binding and higher-order oligomerization of the factor, neuroplastin might become a part of such supramolecular complexes to initiate downstream events of cell signaling. Despite their morphological similarities and the general purpose to sense the environment and facilitate cell-to-cell contact, HEK cell filopodia and neuronal dendritic protrusions serve for different specialized functions. While HEK cell filopodia represent more temporary structures engaged also in cell spreading, dendritic protrusions can become highly specialized structures as they are formed and filled with neuron-specific and membrane-associated and cytosolic proteins which interact with other partners to organize the molecular machinery of the mature spine. As in neurons the extracellular engagement of neuroplastin activates p38 MAPK (Empson et al., 2006), ERK1/2 and PI3 kinase (Owczarek et al., 2010, 2011), these signaling pathways could also be related to homophilic trans-synaptic engagement of Np65 to promote stabilization of the actin cytoskeleton and/or maturation in Shank2-containing protrusions (Boeckers et al., 1999; Sarowar and Grabrucker, 2016). Additionally, the literature recognized TRAF6 as a main upstream activator of the transcriptional factor NFκB pathway (Darnay et al., 1999; Xie, 2013). In young neurons, NFκB activity is not changed by neuronal activity; however, it is necessary for the formation of excitatory synapses during neuronal development (Boersma et al., 2011). Also, the constitutively high NFκB activity in young neurons maintains glutamatergic synapse formation contributing in turn to the establishment of future synapse density in mature neurons (Boersma et al., 2011; Dresselhaus et al., 2018). Future experiments will have to test whether TRAF6 binding to neuroplastin activates NFκB conferring gene expression regulation of synaptic proteins as part of the specialized program for neuronal development.

Although, recent studies have identified neuroplastin as an essential subunit of all four plasma membrane Ca^2+^ ATPases (PMCA1-4) in mature neurons (Herrera-Molina et al., 2017; Schmidt et al., 2017; Gong et al., 2018), we found that elimination of the TRAF6 binding motif of neuroplastin or TRAF6 blockage neither affect the capacity of neuroplastin to interact with nor to promote the expression of PMCA in young neurons or HEK cells (Supplementary Figure S3). These results are discouraging to relate TRAF6-neuroplastin spinogenic function to PMCA in young neurons. Also, PMCA immunoreactivity is rather low in P1-P14 postnatal brains (Kip et al., 2006; Schmidt et al., 2017) and mostly intracellular in young hippocampal neurons (Kip et al., 2006) indicating that PMCA function may not be prominent at early developmental states of neurons. Furthermore, it has been shown that formation of dendritic protrusions does not seem to be triggered by neuronal activity (Verhage et al., 2000; Sando et al., 2017; Sigler et al., 2017), global intracellular calcium transients (Lohmann et al., 2005; Lohmann and Bonhoeffer, 2008) or calcium-dependent signaling in young neurons (Zhang and Murphy, 2004). Certainly, in mature neurons, calcium-dependent signaling plays a critical role in the dynamic and morphology of synaptic spines where one would expect a significant participation of neuroplastin-PMCA complexes.

### TRAF6 Partners Neuroplastin During Synapse Formation

We discovered that neuroplastin and TRAF6 have a spinogenic function operating during a time window in the neuronal development of cultured hippocampal neurons around 6–9 DIV, which is the equivalent time period to the postnatal developmental state of 2–3 weeks-old hippocampus *in vivo* (Dabrowski et al., 2003; Földy et al., 2016). TRAF6 co-localized with and was strictly required by neuroplastin to promote the number of spinogenic dendritic protrusions, which is a critical step for the formation of excitatory synapses. As demonstrated using pharmacological, knock-down, and co-localization approaches, TRAF6 operated to promote the density of postsynaptic protrusions at the same time period that expression of either Np55 or Np65 was effective to rescue the reduced number of dendritic protrusions in *Nptn*^–/–^ neurons. This shows that the TRAF6-dependent mechanism is not essentially dependent on Np65 which, in contrast to Np55, can homophilically interact via its specific trans-adhesive extracellular Ig1 domain (Smalla et al., 2000). This does not rule out the possibility of a later participation of TRAF6 in the trans-stabilization of pre- and post-synapses by extracellular engagement of Np65 (Smalla et al., 2000; Herrera-Molina et al., 2014). Indeed, constitutive elimination specifically of Np65 is not sufficient to alter the synapse density; but was rather reported to cause morphological alterations of hippocampal spines (Amuti et al., 2016). Important in the context of this study is the finding that constitutive elimination of all neuroplastin isoforms, i.e., the absence of Np55 and Np65 reduces the density of excitatory synapses in the hippocampus (Herrera-Molina et al., 2014) whereas that synapse density is not altered in the hippocampus upon induced neuroplastin gene elimination in adult conditional mutant mice (Bhattacharya et al., 2017).

After this time window in the development, neither TRAF6 nor neuroplastin promote dendritic protrusion formation or synapse density. This could be explained by a switch from TRAF6 binding neuroplastin during synaptogenesis in young neurons to binding PSD-95 in mature neurons to promote synaptic plasticity (Ma et al., 2017). Indeed, PSD-95 levels are lower in early synapses than in mature spines (Buckby et al., 2004; Sheng and Hoogenraad, 2007). In mature neurons, TRAF6 binds to PSD-95 and stabilizes the structure of mature synapses and synaptic plasticity (El-Husseini et al., 2000; Ma et al., 2017). The function of TRAF6 in synapse formation is also different from the one reported for the molecule in the embryonic brain, where homozygous deficiency of TRAF6 suppresses programmed cell death induced via p75 neurotrophin receptors-promoted apoptosis (Lomaga et al., 1999; Yeiser et al., 2004). Accordingly, our report unravels a new function of TRAF6 operating in a time window between its functions in the survival of embryonic neurons and in plasticity of mature neurons. Admittedly, the novel spinogenic function of neuroplastin-TRAF6 was identified in primary neuronal cultures, which in general reproduce essential and critical molecular events related to CAMs and synapse formation and maturation (Henderson et al., 2001; Varoqueaux et al., 2006; Williams et al., 2011; Sarto-Jackson et al., 2012; Herrera-Molina et al., 2014; Jiang et al., 2017), and this finding needs to be critically evaluated during the major period of synaptogenesis in the hippocampus *in vivo*. However, the observation that lack of neuroplastin during development leads to a reduced number of excitatory synapses in the hippocampus (Herrera-Molina et al., 2014), a phenotype that cannot be induced by switching off the Nptn gene in adult stages (Bhattacharya et al., 2017), can be taken as an indication that neuroplastin function is required during brain development to determine synapse numbers in this area. Clearly, a verification of the involvement of TRAF6 in this process *in vivo* needs to be tackled in future.

An interesting finding is that the TRAF6 binding motif is not present in other synaptogenic CAMs, suggesting that recruitment of TRAF6 to neuroplastin is a very specific mechanism. CAMs have been proposed as key participants in the regulation of synapse formation and maturation (Henderson et al., 2001; Missler et al., 2003; Varoqueaux et al., 2006; Chubykin et al., 2007; Linhoff et al., 2009; Bozdagi et al., 2010; Robbins et al., 2010). Indeed, CAMs can form specific transmembrane complexes in *cis* that in turn recruit intracellular proteins and activate different spinogenic signaling mechanisms (Yoshihara et al., 2009; Cavallaro and Dejana, 2011; Jang et al., 2017). How can neuroplastin coordinate with other CAM-dependent mechanisms during synapse formation? A compelling study by Földy et al. (2016) used single neuron mRNA sequencing and shown that neuroplastin is highly expressed in excitatory pyramidal neurons in the hippocampus at P7–P14 when massive synapse formation is ongoing (Földy et al., 2016). We interpreted that these high levels of neuroplastin vs. other CAMs during neuronal development may be required by the excitatory neurons to initiate unique and/or distinctive spinogenic mechanisms that other CAMs do not. Our results fit with this idea and with the possibility that neuroplastin may engage with cell-autonomously expressed molecular machineries to promote the formation of a specific group of synapses via regulation of TRAF6-dependent spinogenic signaling (Yoshihara et al., 2009; Jiang et al., 2017; Jang et al., 2017; Sudhof, 2017).

Another interesting finding is that the TRAF6-neuroplastin-dependent spinogenic mechanism induces the formation of a fraction of hippocampal excitatory synapses. Here, we revealed that TRAF6 is critically necessary for the formation of some ∼20–25% of the excitatory synapses while others synapses, including formed excitatory and inhibitory synapses, where unaffected. This was found important for neurons to develop proper excitatory synaptic transmission and neuronal activity. Coincidently, constitutive elimination of neuroplastin gene expression but results in a similar reduction in the number of excitatory synapses accompanied also by decreased synaptic transmission in cultured hippocampal neurons (Herrera-Molina et al., 2014). As neuroplastin depended completely on TRAF6 to promote spinogenesis, it is very likely that the two proteins are promoting the formation of a specific group of excitatory synapses in the hippocampal circuit. Currently, we do not know the specific nature of these particular excitatory synapses, but we suspect that they could be located at the CA1 and/or DG pyramidal neurons as identified in neuroplastin-deficient hippocampus (Herrera-Molina et al., 2014; Bhattacharya et al., 2017).

### Could TRAF6 and Neuroplastin Be Players in Neurological Disorders With Altered Synapse Density?

It is proposed in the field of schizophrenia research that alterations in molecular mechanisms responsible for synapse architecture and/or density would impact on the pathogenesis of this disorder (Boda et al., 2010; Caldeira et al., 2019). As, reduced synapse density (Caldeira et al., 2019) and increased TRAF6 mRNA expression were demonstrated in the hippocampus and striatum of schizophrenic patients^2^, it is tempting to speculate that altered timing or levels of TRAF6 expression could contribute to an impairment in synapse formation – a hypothesis that needs to be tested. Elucidation of this matter may also contribute to the understanding of the association of neuroplastin expression with schizophrenia risk (Saito et al., 2007, see footnote 2).

## Data Availability Statement

The raw data supporting the conclusions of this article will be made available by the authors, without undue reservation.

## Author Contributions

SV and AM conducted most of the experiments and raw data analysis. LJ conducted the PMCA experiments. A-CL and RR made and characterized constructs. JH conducted the SPR experiments. RM conducted the *in silico* modeling. MP conducted the Patch Clamp experiments. RH-M supported the experiments, conducted the MEA and Patch Clamp experiments, and wrote the manuscript draft. RH-M, MK, MP, MN, CS, and EG contributed to the experimental design and data interpretation. All the authors contributed to the manuscript’s final version.

## Conflict of Interest

The authors declare that the research was conducted in the absence of any commercial or financial relationships that could be construed as a potential conflict of interest.

## Footnotes

1 http://elm.eu.org/

2 http://www.szdb.org/SZDB/
