# Supplementary material for: The Interaction of TRAF6 With Neuroplastin Promotes Spinogenesis During Early Neuronal Development
Source: Front Cell Dev Biol. 2020 Dec 9;8:579513. doi: 10.3389/fcell.2020.579513 (PMC7755605; doi:10.3389/fcell.2020.579513)
Supplement: Supplementary file 1 [file Data_Sheet_1.pdf]

## Supplementary Figures and Figure Legends

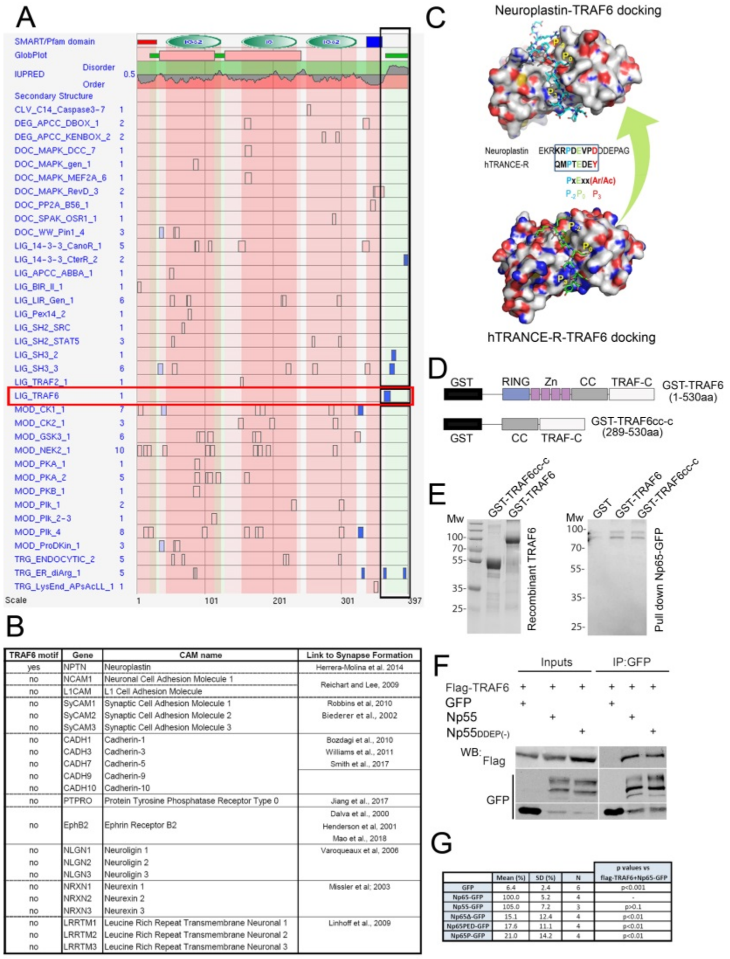


**Fig. S1. Search for TRAF6 binding motifs in synaptogenic cell adhesion molecules and characterization of the binding motif in neuroplastin.**

**A.** A Database search for TRAF6 binding sites revealed one motif in the cytoplasmic C-terminal region of neuroplastin. ELM database (http://elm.eu.org/) read out table shows identified binding motifs in the mouse neuroplastin structure (top drawing). The three extracellular Ig-like domains are depicted in green, transmembrane domain in blue and intracellular tail is not colored. TRAF6 binding motif (blue square in the red horizontal frame) is identified in the cytoplasmic tail (black vertical frame).

**B**. List synaptogenic cell adhesion molecules analyzed using the ELM database. Note that only neuroplastin was identified to contain a TRAF6 binding motif.

**C**. Modeling neuroplastin-TRAF6 binding. The docking of TRAF6 to neuroplastin is based on the hTRANCE-R-TRAF6 interaction according to provided structures (Ye et al., 2002). The local peptide docking of the tail of neuroplastin (cyan) is shown on the top and the hTRANCE-R template complex is shown below (green). Protein structure

similarity (TM-score) = 0.991, Interaction similarity = 108.0, and Estimated accuracy = 0.868. Positions $P_{-2}$, $P_0$ and $P_3$ in the TRAF6 motif are indicated in both peptide-protein complexes. Protein surfaces are colored based on element (C in white; O in red; N in blue; S in orange).

**D.** Representation of GST-TRAF6 and GST-coiled coil-TRAF-C domain ($TRAF6_{cc-c}$) recombinant proteins. RING domain, zinc fingers (Zn), coiled-coil region (CC) and C-terminal domain (TRAF-C) are indicated.

**E**. Left: Coomassie gel showing recombinant GST-TRAF6 and GST-$TRAF6_{cc-c}$ proteins. Right: Western blot showing that both recombinant proteins are pulled-down by Np65-GFP from total extracts of transfected HEK cells.

**F**. Co-precipitation of TRAF6 with neuroplastin 55 from HEK cells homogenates. The two Np55 isoforms (with and without DDEP insert) are effective to co-precipitate TRAF6. HEK cells were transfected with the indicated constructs, left to express the tagged proteins for 24 hours, and lysed with RIPA lysis buffer. The extracts were immunoprecipitated with anti-GFP antibody coupled to magnetic beads. Precipitated complexes were resolved by SDS-PAGE and immunoblotted with anti-Flag or anti-GFP antibodies. Representative images from 3 independent experiments.

**G**. Quantification of the intensity of TRAF6-flag bands resulting from co-precipitation with the different neuroplastin constructs from HEK cell homogenates. Note that TRAF6-flag co-precipitated with Np65-GFP is normalized to 100% and served as reference to compare the other conditions as indicated. Significantly decreased TRAF6-flag signal is detected after elimination of TRAF6 binding site in Np65. p values were obtained using Mann-Whitney test.

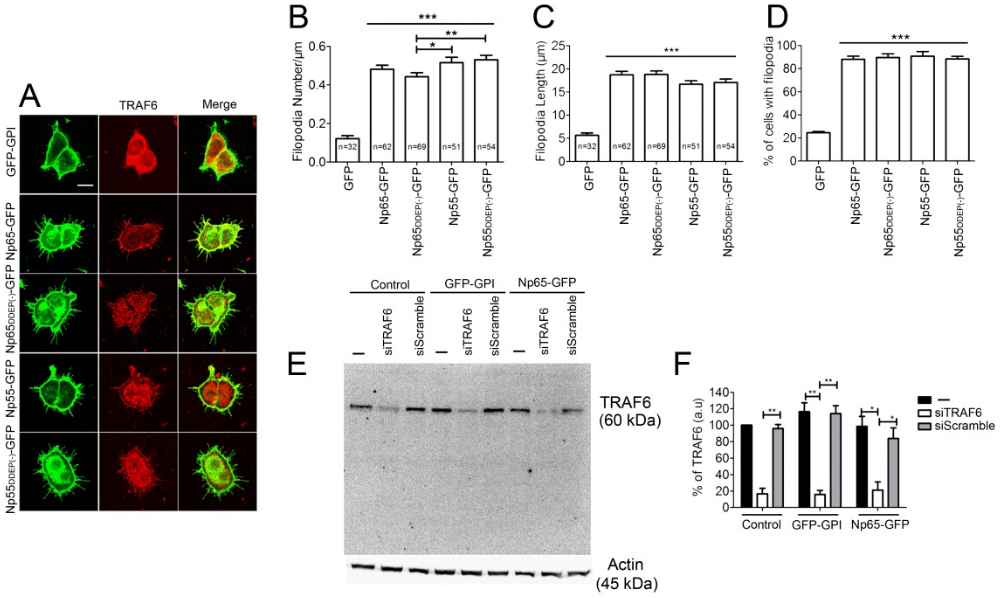


**Fig. S2. HEK cell filopodia formation and TRAF6 recruitment by neuroplastin isoforms. Characterization of TRAF6 knockdown efficiency.**

**A**-**D**. All four splice variants of neuroplastin are similarly robust to promote translocation of endogenous TRAF6 to the cell membrane and to increase both number and length of filopodia. (**A**) Confocal images displaying representative examples of HEK cells transfected with different neuroplastin constructs and stained for TRAF6 as for Figure 2D. Scale bar=10 µm. (**B**) Number of filopodia (GFP=0.12 ± 0.01 N=32; Np65-GFP=0.48 ± 0.02 N=62; $Np65_{DDEP(-)}$-GFP=0.44 ± 0.02 N=69; Np55-GFP=0.52 ± 0.03 N=51; $Np55_{DDEP(-)}$-GFP=0.53 ± 0.02 N=54) (**C**) Filopodia length (GFP=5.66 ± 0.49; Np65-GFP=18.72 ± 0.77; $Np65_{DDEP(-)}$-GFP=18.79 ± 0.76; Np55-GFP=16.68 ± 0.76; $Np55_{DDEP(-)}$-GFP=17.08 ± 0.72) and (**D**) Percentage of cells with filopodia are displayed as mean ± SEM. Student's t-test (**B**,**C**) or Mann-Whitney test (**D**) were applied. *$p<0.05$, **$p<0.01$, and ***$p<0.001$ vs GFP.

**E**, **F**. Assessment of TRAF6 knockdown efficiency upon siRNA treatment. (**E**) Total cell homogenates were resolved by SDS-PAGE and immunoblotted for endogenous TRAF6 with a mouse anti-TRAF6 antibody. Actin was detected to control protein loading. (**F**) The graph shows the densitometric quantification of TRAF6 bands from three independent experiments. **$p<0.01$ or *$p<0.05$ using Mann-Whitney test.

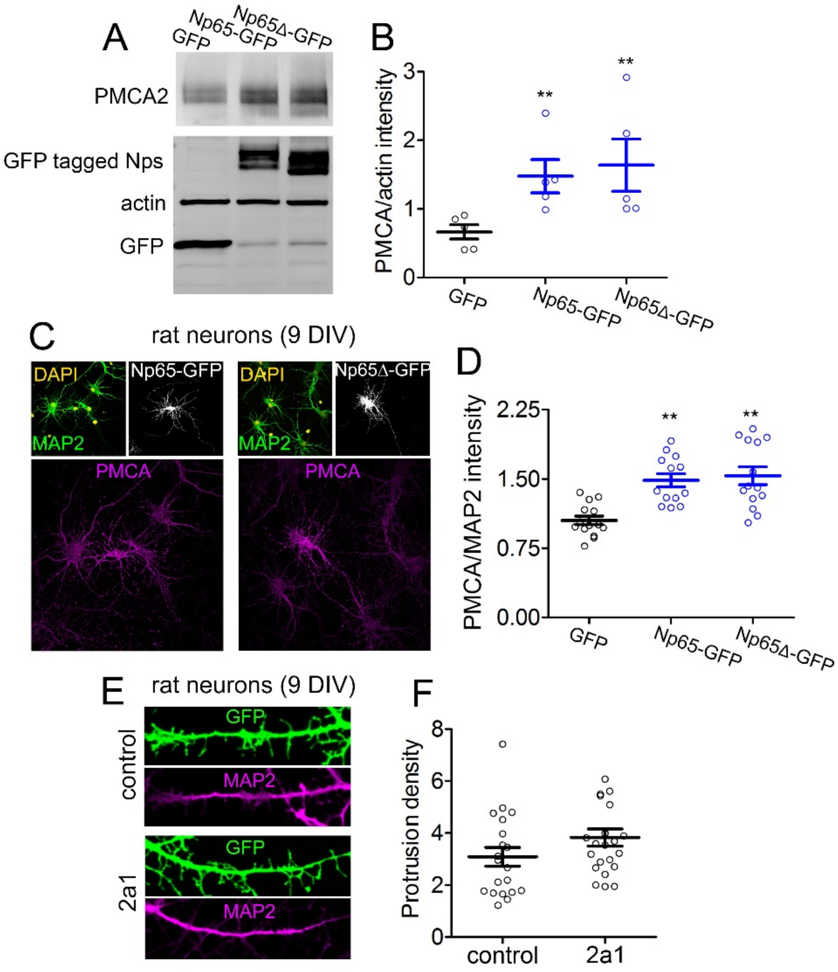


**Fig. S3. PMCA is not essential in TRAF6-neuroplastin promoted dendritic protrusion formation.**

**A**, **B**. Protrusion formation does not depend on neuroplastin-PMCA interaction. (**A**) Np65-GFP and Np65Δ-GFP equally increase total PMCA2 levels in HEK cells. Cells were transfected with the indicated constructs, harvested 24 hours later and lysed with RIPA lysis buffer. Western blot analysis shows that levels of PMCA2 are increased upon co-transfection with Np65-GFP and with Np65Δ-GFP as indicated. Detection of actin is used to control for equal loading. (**B**) Quantification of PMCA2 levels are normalized to actin using data from five independent experiments. $^{**}p<0.01$ vs. GFP using Mann-Whitney test.

**C**, **D**. Np65-GFP and Np65Δ-GFP lacking the intracellular TRAF6 binding domain are equally effective to increase PMCA expression in young hippocampal neurons. (**C**) At 7DIV hippocampal neurons were transfected with plasmids encoding Np65-GFP or Np65Δ-GFP. At 9DIV, neurons were fixed and stained with anti-MAP2 and anti-pan-

PMCA antibodies. Scale bar=10 µm. (**D**) Quantification of the intensity of PMCA immunofluorescent signal normalized to MAP2 signal using data from three independent cultures. $^{**}p<0.01$ vs. GFP using Student's t-test. (GFP=1.05 ± 0.04 N=14; Np65-GFP=1.48 ± 0.07 N=13; Np65Δ-GFP= 1.53 ± 0.09 N=14).

**E**, **F**. Hippocampal neurons were transfected with GFP-expressing plasmid at 7 DIV. Next day, neurons for were treated with the PMCA inhibitor Caloxin 2a1 for 24 hours. After fixation and immunostaining with anti-GFP antibody and anti-MAP2 antibody followed by proper secondary antibodies, neurons were imaged using a confocal microscope with a 63X objective under 3X digital zoom factor. (**F**) Protrusion density in control and Caloxin 2a1-treated neurons from two independent cultures (control=3.08 ± 0.35 N=20; 2a1= 3.83 ± 0.39 N=25).

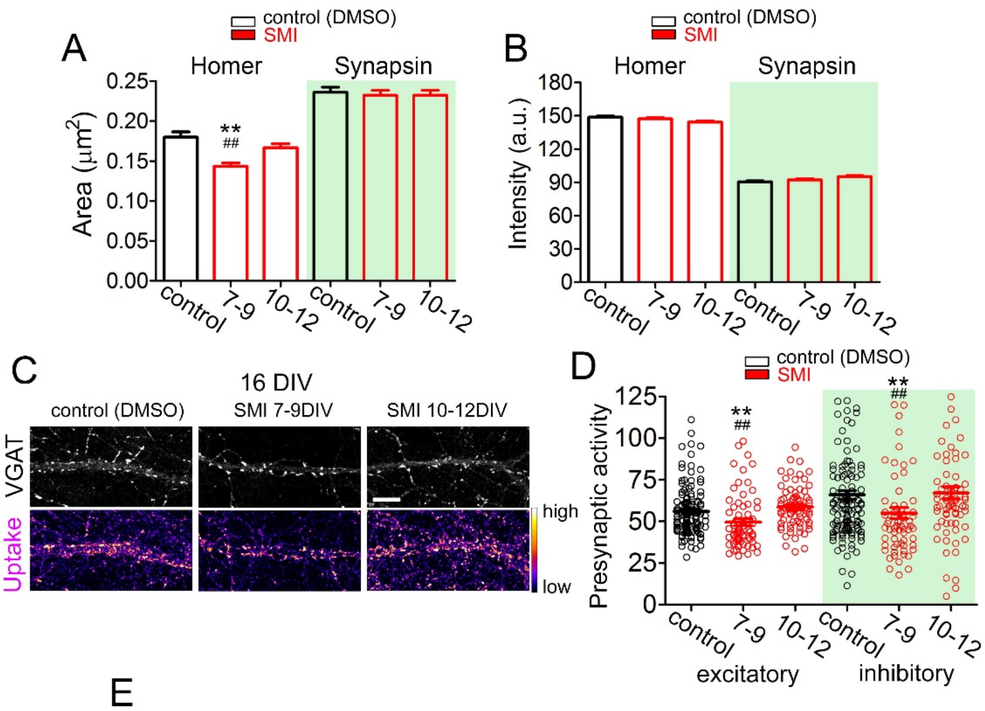


E

| presynapse: | condition | area of vesicle release (area of puncta) | P value vs. control | activity level (intensity per pixel) | P value vs. control |
|---|---|---|---|---|---|
| excitatory | control | 0.3924 ± 0.009 N=130 | | 138.3 ± 0.56 N=130 | |
| | SMI 7-9 | 0.3446 ± 0.012 N=67 | p<0.001 | 139.8 ± 0.99 N=67 | p>0.05 |
| | SMI 10-12 | 0.4011 ± 0.009 N=69 | p>0.05 | 140.3 ± 0.87 N=69 | p>0.05 |
| inhibitory | control | 0.5520 ± 0.012 N=130 | | 114.7 ± 2.8 N=130 | |
| | SMI 7-9 | 0.5535 ± 0.019 N=67 | p>0.05 | 100.2 ± 5.062 N=67 | p<0.01 |
| | SMI 10-12 | 0.5574 ± 0.017 N=69 | p>0.05 | 113.8 ± 5.0 N=69 | p>0.05 |

**Fig. S4. TRAF6 in synapse formation and network-driven synapse activity.**

**A**, **B**. SMI TRAF6 from 7 to 9 DIV, but not from 10 to 12 DIV, reduces the number of excitatory synapses. (**A**) Quantification of the area (Homer: control=0.172 ± 0.004; 7-9=0.143 ± 0.004; 10-12=0.162 ± 0.006. Synapsin: control=0.236 ± 0.006; 7-9=0.232 ± 0.007; 10-12=0.248 ± 0.003) and (**B**) fluorescence intensity (Homer: control=148.58 ± 0.99; 7-9=147.13 ± 0.99; 10-12=145.58 ± 1.12. Synapsin: control=90.37 ± 0.79; 7-9=92.21 ± 0.84; 10-12=96.77 ± 2.01) of homer- and synapsin-positive puncta. For Homer, **p<0.01 vs. control and $^{\#\#}$p<0.01 vs. 10-12 using Student's t-test.

**C**, **D**. Reduced presynaptic vesicle recycling in SMI TRAF6-treated 16 DIV-old neurons. (**C**) Dendritic segments of rat neurons were treated with SMI TRAF6 as indicated and tested for uptake of fluorescently labelled antibody against the luminal part of the presynaptic protein synaptotagmin (see Material and Methods). Anti-VGAT antibody was used to stain inhibitory presynapses. Photomicrographs with a confocal microscope are shown. The heat scale bar indicates uptake levels. Scale bar=10 μm. (**D**) Quantification of presynaptic activities from three independent experiments

(Excitatory: control=55.97 ± 1.28 N=130; 7-9=49.58 ± 2.025 N=67; 10-12=58.73 ± 1.568 N=69; Inhibitory: control=66.00 ± 2.63 N=130; 7-9=54.99 ± 3.30 N=67, 10-12=67.11 ± 3.62 N=69). **p<0.01 vs. control and ##p<0.01 between synapse type using Student's t-test.

**E**. Statistical breakdown of the activities in inhibitory and excitatory presynapses shown in C and D.
